# Dynamics-function relationship in the catalytic domains of N-terminal acetyltransferases

**DOI:** 10.1016/j.csbj.2020.02.017

**Published:** 2020-03-03

**Authors:** Angèle Abboud, Pierre Bédoucha, Jan Byška, Thomas Arnesen, Nathalie Reuter

**Affiliations:** a Department of Informatics, University of Bergen, Bergen, Norway; b Computational Biology Unit, Department of Informatics, University of Bergen, Bergen, Norway; c Faculty of Informatics, Masaryk University, Brno, Czech Republic; d Department of Biological Sciences, University of Bergen, Bergen, Norway; e Department of Biomedicine, University of Bergen, Bergen, Norway; f Department of Surgery, Haukeland University Hospital, Bergen, Norway; g Department of Chemistry, University of Bergen, Bergen, Norway

**Keywords:** Acetylation, N-terminal acetyltransferases, Protein dynamics, Normal modes analysis, Ligand specificity

## Abstract

N-terminal acetyltransferases (NATs) belong to the superfamily of acetyltransferases. They are enzymes catalysing the transfer of an acetyl group from acetyl coenzyme A to the N-terminus of polypeptide chains. N-terminal acetylation is one of the most common protein modifications. To date, not much is known on the molecular basis for the exclusive substrate specificity of NATs. All NATs share a common fold called GNAT. A characteristic of NATs is the β6β7 hairpin loop covering the active site and forming with the α1α2 loop a narrow tunnel surrounding the catalytic site in which cofactor and polypeptide meet and exchange an acetyl group.

We investigated the dynamics-function relationships of all available structures of NATs covering the three domains of Life. Using an elastic network model and normal mode analysis, we found a common dynamics pattern conserved through the GNAT fold; a rigid V-shaped groove formed by the β4 and β5 strands and splitting the fold in two dynamical subdomains. Loops α1α2, β3β4 and β6β7 all show clear displacements in the low frequency normal modes. We characterized the mobility of the loops and show that even limited conformational changes of the loops along the low-frequency modes are able to significantly change the size and shape of the ligand binding sites. Based on the fact that these movements are present in most low-frequency modes, and common to all NATs, we suggest that the α1α2 and β6β7 loops may regulate ligand uptake and the release of the acetylated polypeptide.

## Graphical abstract

**Figure:**
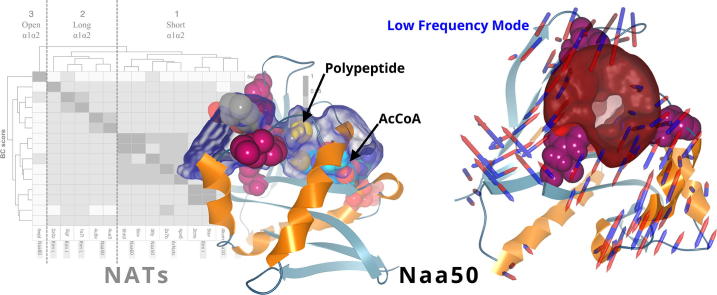

## 1 Introduction

Acetyltransferases are enzymes catalysing the transfer of an acetyl group from the co-factor acetyl-coenzyme A (Ac-CoA) to a substrate. Among them, Nα-terminal acetyltransferases (NATs) perform N-terminal acetylation of polypeptide chains. NATs acetylate 80–90% of the proteins of the human proteome [1] and N-terminal acetylation has been shown to play a role in various biological processes from protein folding to gene regulation [2]. Dysregulation or mutations of NATs have been linked to several diseases including tumour development [2], [3], [4], [5] and initiatives are already undertaken to develop inhibitors targeting the relevant NATs [6].

**Fig. 1:**
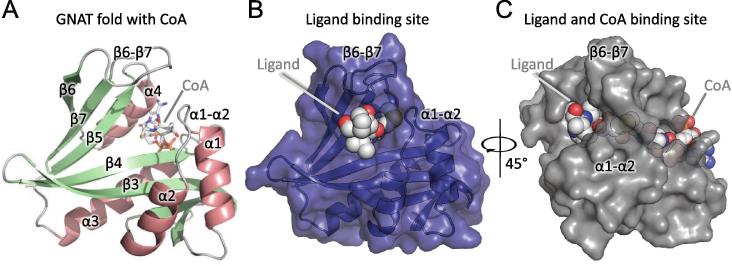
GNAT fold and substrate binding sites in NAT catalytic domains. (A) Cartoon representation of the GNAT fold of human Naa50 (PDB ID: 3TFY). It consists of 6 β-strands (green) and 4 α-helices (salmon pink) organised in the following topology: B1-H1-H2-B2-B3-B4-H3-B5-H4-B6. The Ac-CoA represented in sticks with its backbone in grey sits between helix α4 and the β4α3 loop. (B) Human Naa50 (cartoons and blue solvent-accessible surface) bound to substrate Met-Leu-Gly (van der Waals spheres) (PDB ID: 3TFY). (C) Naa60 (grey solvent-accessible surface) bound to a bisubstrate CoA-Ac-Met-Lys-Ala-Val (PDB ID: 5ICV). Loops β6β7 and α1α2 are labelled in (B) and (C). (For interpretation of the references to color in this figure legend, the reader is referred to the web version of this article.)

Most acetyltransferases share the GNAT fold (Gcn5-related N-acetyltransferases) [7]. It consists of a three-layered αβα sandwich containing seven β-strands and four α-helices (Fig. 1A). The GNAT fold displays two features that are conserved through most of the acetyltransferases and all the NATs, and are related to the transfer of an acetyl to an amino group. The first is a conserved sequence motif essential for Ac-CoA binding (Q/RxxGxG/A) and located on the turn between strand β4 and helix α3 [7], [8], [9]. Interestingly, Rathore et al. reported an extended version of this motif (Q/RxxGxG/AxxL) in a recent study where they could also determine that the diversification of NATs occurred before the evolution of eukaryotes [10]. The β4 strand together with β4-α3 and α4 form much of the Ac-CoA binding site. The second salient feature of the GNAT fold is the V-shaped configuration of the two parallel strands β4 and β5, forming a groove where the extremities of the Ac-CoA and of the substrate peptide meet, positioning the acetyl group and the amino group close enough for the catalytic reaction to occur [7].

Substrate specificity varies drastically within the NATs, offering a palette of enzymes able to target a large spectrum of N-terminal sequences. As of now, eight NATs (NatA-NatH) have been identified in eukaryotes [2], [11], [12], three in prokaryotes (RimI, RimJ and RimL) [13], [14] and one in archaea (referred here as ArNat) [15]. NATs are classified based on their composition and substrate specificity. In eukaryotes the catalytic subunits are named Naa10, Naa20, etc. to Naa80 (Cf. Table 1 and Ref. [16]). The β-hairpin β6β7, the α1α2 loop and the helix α2 contain amino acids forming the boundary of the substrate binding pocket. NAT substrates are positioned so that two to three residues are in the peptide binding site [17], [18], [19], [20]. Two conserved tyrosines located on the β6β7 loop and another one on the α1α2 loop have been shown to interact with the substrate backbone via hydrogen bonds [21]. Both loops cover the groove containing the catalytic site (Fig. 1B) to which substrate peptides bind. The access to the catalytic site is very tight in NATs as it is shielded by the β6β7 hairpin loop that forms a tunnel together with the α1-α2 region (Fig. 1C). Using relatively short molecular dynamics simulations of the human Naa10 and Naa50, we earlier observed that (i) the flexibility of helix α2 changes upon ligand binding in Naa50 [21] and (ii) the β6β7 hairpin loop is highly mobile in both enzymes [22].

**Table 1:** N-terminal acetyltransferases (NATs) found in PROSITE, Uniprot and PDB databases.

Group	Super Kingdom / Genus	E.C number	Uniprot	PDB ID	Chain
ArNat	Archaea	2.3.1.-			
	*Sulfolubus*		Q980R9	**2x7b**[88], 4lx9[36], 4r3k[34], 4r3l[34], 5c88[89]	A, A, A, A, A
	*Thermoplasma*		Q97CT7	**4pv6**[90]	A
Naa10	Eukaryota	2.3.1.255			
	*Schizosaccharomyces*		Q9UTI3	**4kvm**[18], 4kvo[18], 4kvx[18]	E, E, B
	*Saccharomyces*		P07347	4xnh, 4xpd, 4y49, 4hnw, 4hnx, 4hny	B, B, B, B, B, B
Naa20	Eukaryota	2.3.1.254			
	*Candida*		C4YDZ9	5k04[53], **5k18**[53]	B, B
Naa40	Eukaryota	2.3.1.257			
	*Schizosaccharomyces*		Q9USH6	**4ua3**[20]	A
	*Homo sapiens*		Q86UY6	**4u9v**[20], 4u9w[20]	A, B
Naa50	Eukaryota	2.3.1.258			
	*Homo sapiens*		Q9GZZ1	2ob0, 2psw, **3tfy**[17], 4x5k	A, A, C, A
Naa60	Eukaryota	2.3.1.259			
	*Homo sapiens*		Q9H7X0	5hgz[19], **5hh0**[19], 5hh1[19], **5icv**[55], 5icw[55]	A, A, A, B, A
Naa80	Eukaryota	–			
	*Drosophila*		Q59DX8	**5wjd**[37], 5wje[37]	
RimI	Bacteria	2.3.1.128			
	*Salmonella*		Q8ZJW4	2cnm[56], **2cns**[56], 2cnt[56]	A, A, A
	*Escherichia*		P0A946	**5isv**	A
RimJ	Bacteria	2.3.1.128			
	*Aliivibrio*		Q5DZH6	**3igr**	A
RimL	Bacteria	2.3.1.128			
	*Salmonella*		Q8ZPC0	1s7f[91], 1s7k[91], **1s7l**[91], 1s7n[91], 1z9u	A, A, A, A, B
	*Thermus*		Q5SHD1	**2z0z**[92], 2zxv[92]	A, A
			Q72HN8	2z10[92], 2z11[92]	A, A

Representative structures chosen for each Uniprot code are highlighted with a bold PDB ID. They form our dataset of 15 structures, augmented by 19 structures (PDB IDs written with regular black fonts) to form the dataset of 34 structures used for the RMSD analysis. Two representatives were selected for the Naa60 group as they have different topologies (5hh0 contains one extra helix at the C-terminus). PDB IDs written in light grey are available structures that are not included in our dataset due to poor quality (see Section 4). One chain was selected for the calculations. For information on the dataset preparation the reader is referred to the methods. Q5SHD1 and Q72HN8 are ribosomal-protein-alanine acetyltransferases from two different strains of Thermus Thermophilus but they are identical in sequence, hence we chose only one representative for the two Uniprot IDs.

Enzyme dynamics is important for their function, with catalytic residues – unlike ligand binding sites – being placed at rigid positions of the fold. It has also been shown that functionally relevant flexibility is conserved between enzymes sharing the same fold [23], [24], [25]. Normal mode analysis (NMA) using elastic network models (ENM) is an efficient computational method that has proven reliable to characterize the flexibility intrinsic to protein structures [26], [27], [28], [29], [30], [31], [32]. It has also been successfully used to conduct comparative analyses of multiple protein structures [23], [26].

In this study we use an elastic network model and normal mode analysis to characterize the intrinsic dynamics of all known NATs catalytic domains. We perform a comparative analysis of their low-frequency normal modes and uncover a dynamics pattern intrinsic to the GNAT fold. It consists in correlated movements of two subdomains, one on either side of the β4β5 V-shaped split. The β6β7 loop follows the movements of the C-terminal subdomain. We investigate how the movements of β6β7 and the rest of the protein modify the ligand binding sites and show how they influence the access route of the ligand to the co-factor and catalytic site.

## 2 Results

Our dataset consists of 15 distinct proteins and represents ten types of NATs defined according to their composition and substrates (S1 and S2 Tables). The 15 structures are extracted as a non-redundant dataset of 34 structures that are all listed in Table 1 with their respective PDB ID. The dataset spans the three domains of Life, where six out of ten NATs stem from eukaryotes (NatA, NatB, NatD, NatE, NatF, NatH), three from bacteria (RimI, RimJ and RimL) and one from archaea (ArNat). In what follows, the ten types of NATs found in our dataset are referred to by the name of the catalytic subunit of each NAT complex (Naa10, Naa20, etc… reported in Table 1 in the column titled “Group”).

### 2.1 The GNAT fold and accessory structural elements

**Fig. 2:**
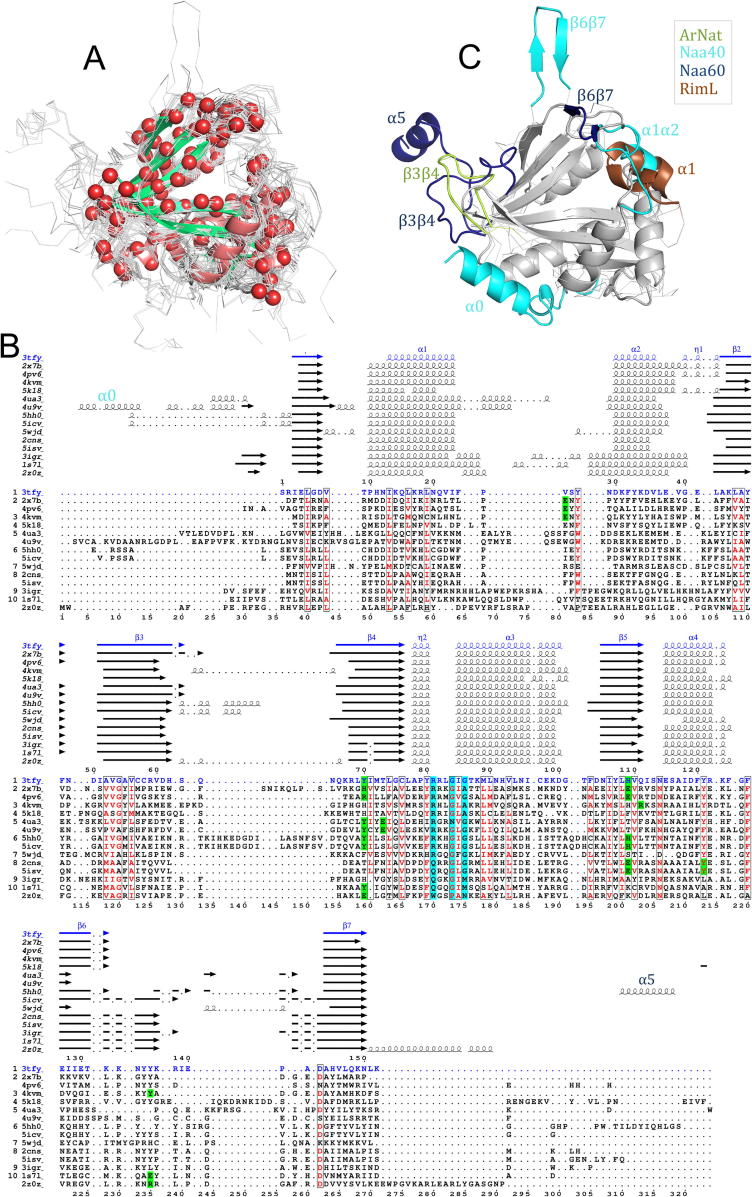
Structural alignment of Naa representatives. (A) MUSTANG structural alignment of all NAT structures listed in Table 1. The backbone of each structure is represented with lines except for that of the reference structure Naa50 (PDB ID: 3TFY), which is represented with cartoons. The red beads represent the 116 aligned C-alpha atoms and are mostly located on the common structural elements of the GNAT fold. The orientation is similar to that of the structures on panel C. (B) Multiple sequence alignment resulting from the structural alignment. Naa50 sequence is written with blue fonts, Ac-CoA-binding motifs are highlighted with cyan boxes and residues involved in the catalytic activity with green boxes. Sequences are labelled with the PDB ID from which their secondary structure elements are retrieved. The image results from the use of ESPript [87]. (C) Cartoon representation of the shared GNAT fold (in grey) and structural variations: helix α0 in Naa40, helix α5 in Naa60, long β6β7 loops in Naa40 and Naa60, long α1α2 loops in Naa40 and RimL, long β3β4 loops in ArNats and Naa60. The orientation is similar to that of the superimposition on panel A. (For interpretation of the references to color in this figure legend, the reader is referred to the web version of this article.)

We aligned all structures in the dataset using the multiple structure alignment tool MUSTANG [33] (see Section 4). The structure of Naa50 (PDB ID: **3TFY**) was used as reference. The alignment led to 119 C-alpha atoms’ positions conserved (Fig. 2A). While sequence similarity between pairs of NATs is relatively low (23% identity on average), the secondary structure elements of the GNAT fold align well (Fig. 2B). The region between the end of strand β4 and helix α4 is the most conserved sequence-wise. It contains several of the amino acids involved in catalysis (located on strands β4 and β5), as well as the Ac-CoA binding motif (R/QxxGxG/A on β4-α3) (Fig. 2B). Noticeably, position 218 of the alignment (Asn114 in Naa50) is an asparagine conserved through all the 14 NATs, except in Naa80 where it is replaced by an aspartate (Asp 127) (Fig. 2B). In all the structures this residue sits close to the Acetyl-CoA oxygen of the pantothenic acid (the oxygen from the carbonyl linking to the mercapto-ethylamine) [34]. Structural differences between NATs are restricted primarily to the N- and C-terminal regions, before helix α2 and after strand β6, respectively. The differences stem either from longer elements of the GNAT domain or from additional accessories. The latter are secondary structure elements that are not part of the GNAT fold such as the N-terminal helix in Naa40, referred to as helix α0. Another such addition to the GNAT fold is a sixty-one amino acid-long segment at the C-terminal end of Naa60. The positions of only the first thirty amino acids of this extension are resolved in the X-ray structure (see dark blue segment on Fig. 2C) and show the presence of one α-helix (α5). Secondary and tertiary structure prediction from sequence indicate the presence of an additional α-helix which, with α5 anchors the protein to the Golgi membrane [35].

**Fig. 3:**
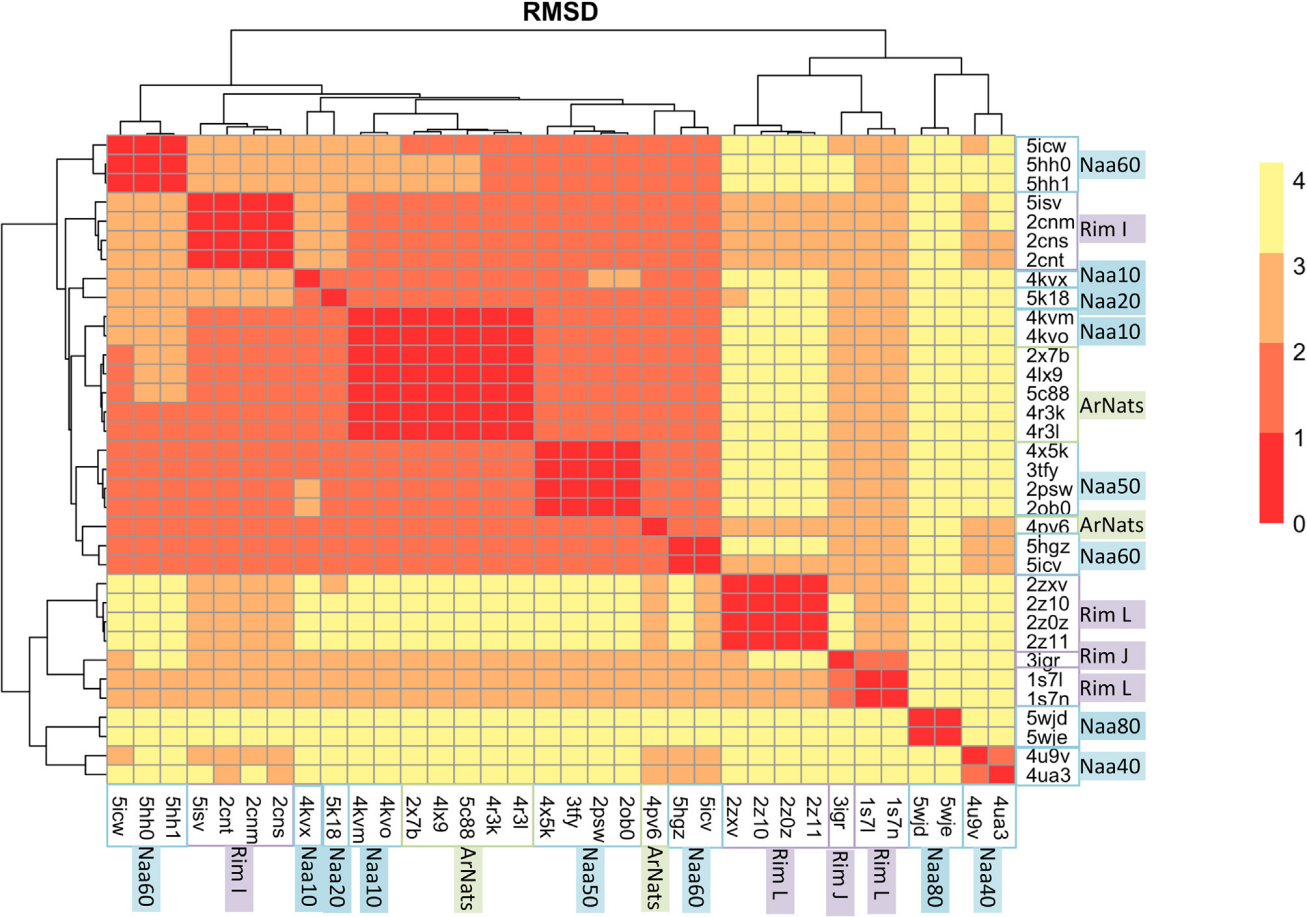
Heatmap representation of the pairwise Root Mean Square Deviations (RMSD). The dendrogram reflects the hierarchical clustering based on the RMSD values. The heatmap color scale goes from red (0 Å < RMSD < 1 Å; structural similarity) to yellow (3 Å < RMSD < 4 Å). Names of enzymes from eukaryotes are highlighted in blue, those of bacteria in purple and green is used for archaeal NATs. (For interpretation of the references to color in this figure legend, the reader is referred to the web version of this article.)

We quantified the structural similarity between Naas by calculating pairwise root mean square deviations (RMSD) between the thirty-four structures of the dataset. All values are shown on a heatmap (Fig. 3). As expected, RMSD values between structures belonging to the same group are small, which is in agreement with the fact that structures within a group are orthologues or structures of the same protein but in different forms (e.g. apo vs. holo) (Cf. Table S1). Alignment of a representative dataset consisting of only one structure per Uniprot accession number (Table 1) yields comparable RMSD values (Fig. S1).

Hierarchical clustering of the structures according to the pairwise RMSD reveals two main groups. The first group consists of: ArNats, Naa10, Naa20, Naa50, Naa60, RimI. In this cluster, the closest structures are the archaeal Naas and the eukaryotic Naa10 with RMSD values of up to 1.2 Å. The RMSDs between ArNats (e.g. 4lx9) and Naa10, Naa50, Naa60 and the RimI are lower than 2 Å. The structural proximity of the archaeal Naa with enzymes belonging to other groups is in agreement with what is known about its substrate specificity. The archaeal Naa from *Sulfolobus* uses two different catalytic strategies; it can acetylate either serines, like Naa10 enzymes, or methionines, like Naa50 enzymes (S2 Table). Mutations of key residues from the α1α2 loop were shown to shift the substrate specificity from small amino acids to methionines [36]. In this study, Liszczak et al. suggested these mutations as part of a model of the evolution of a eukaryotic ancestor to a more diverse family with different substrate specificity. The second main group consists of three clusters Naa40, Naa80 and the bacterial RimJ and RimL, which appear to be the most structurally distant from other structures in the dataset with RMSD values between 2.5 and 3.9 Å. They are composed of longer elements in the GNAT fold that influence the orientation of the secondary structure moving them further away from the other NATs. As shown in Fig. 2B the entire region from α1 to α2 is longer in RimL than in other Naas (6, 4 and 7 additional residues for helices α1, α2 and α1α2 loop, respectively). Naa40 also has an extended α1 helix of eight amino acids and an extra N-terminal helix α0 consisting of 17 amino acids. This α0 helix sits under the GNAT fold and changes the topology of the region β1-α2. The α1α2 loop and the longer α1 helix cover the active site and the β6β7 hairpin loop is flanked away from the active site (Fig. 2C). The structure of the β6-β7 region in Naa80 is different from that of typical NATs. It has a shorter β6-strand, which leads to a different orientation of the β6β7 loop and a ligand binding site opened more widely than in the other NATs [37].

The structural alignment of the existing NATs structures obtained in this first step builds the premises for the comparative dynamics analysis. Such an analysis is indeed reliant on a good quality sequence alignment of the studied structures [26]. Moreover, this first step of our study highlights the importance of the GNAT fold as a framework for co-factor and ligand binding and illustrates the fine-tuning achieved by the additional structural elements which contribute to the functional diversity of NATs.

### 2.2 Comparative dynamics analysis of NATs

**Fig. 4:**
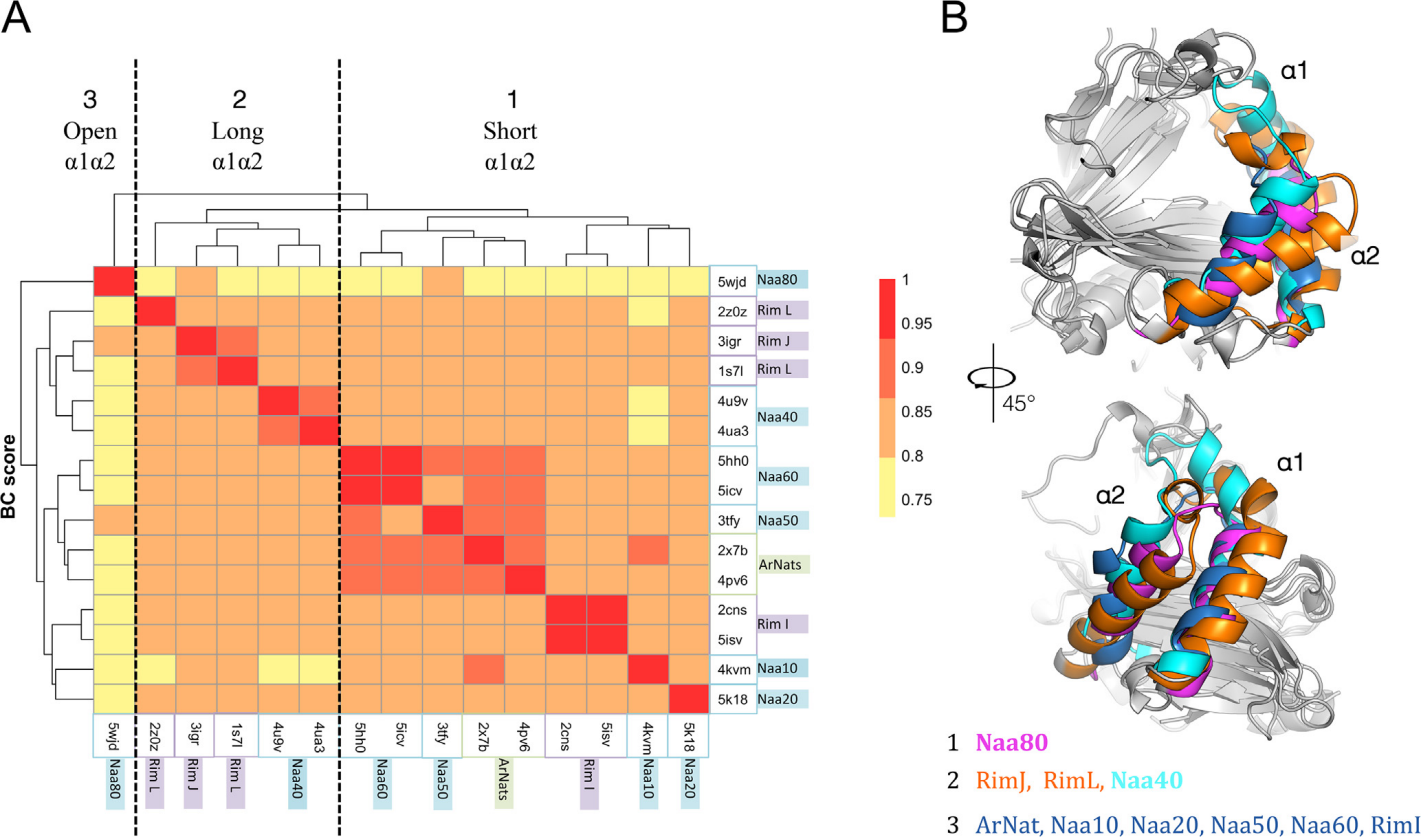
Comparison of the dynamics of the NATs using the Bhattacharyya score (BC score) on a non-redundant dataset. (A) Heatmap representation of the pairwise BC score between the representative structures (see Section 4). The color scale of the BC score goes from red for high similarity in intrinsic dynamics to yellow for higher dissimilarity. The dendrogram is the hierarchical clustering of the pairwise BC score. The names of the groups are written in boxes colored in blue for eukaryotes, purple for bacterial and green for archaeal NATs. (B) Cartoon representation of the structures aligned and used to calculate the pairwise BC score. The helices α1 and α2 are colored according to the cluster they belong to (see color of boxes on the axes of the dendrogram). The first cluster composed of the archaeal Naa, Naa10, Naa20, Naa50, the Naa60 and the RimI (colored in dark blue) shares a shorter helix α1 than that of the second cluster consisting of Naa40 (colored in cyan), RimJ and RimL (colored in orange). Naa80 (colored in magenta) separates from the others; it has the shortest α1α2 loop and the widest binding site of all NATs. (For interpretation of the references to color in this figure legend, the reader is referred to the web version of this article.)

The Bhattacharyya score (BC score) quantifies the intrinsic dynamics (dis)similarity between each pair of aligned cores of proteins in a dataset [38]. Prior to calculating the BC score the structures of the representative dataset were superimposed to generate a structure-based sequence alignment and the aligned cores are shown on Fig. 2A. We then calculated the normal mode of each of the structures in the dataset. All were modelled using an elastic network. We then calculated the BC scores between each pairs of proteins (Table 1). A heatmap representation of the BC values is shown in Fig. 4A, together with a dendrogram representing the clustering. The values of the pairwise BC scores are all high indicating a high degree of similarity in flexibility. Yet there are also differences between the structures which are clustered in three main groups containing (1) Naa10, Naa20, Naa50, Naa60, archaeal NATs, bacterial RimI; (2) bacterial RimJ and RimL, as well as Naa40 which is the only eukaryotic NAT in this group, and (3) Naa80.

The structural difference between structures in three identified clusters is mostly in their N-terminal region. This is a strong suggestion that the dynamics of this region, or the influence of this region on the dynamics of the overall structures, explain the difference in intrinsic dynamics between the three groups. Yet, the RMSD between aligned Cα positions does not lead to the same clustering of structures (Cf. heatmap on Fig. S1), indicating that the structure of the core GNAT structural elements is not playing a role in the BC clustering, but rather the effect of the non-conserved structural elements on the fold dynamics is. The GNAT fold has a region of high variability from the N-terminal to the α2 helix [7] (Fig. 2C and 4B).

Interestingly these clusters correlate fairly well with the specificity of the enzymes in each group. The first group contains the Naas acetylating methionine: ArNats, Naa50 and Naa60 and the one acetylating preferentially small residues: Naa10 and RimI, respectively (S2 Table). Naa20 acetylates methionines followed by acidic residues and is clustered with Naa10. The latter has also been shown to shift substrate specificity towards acidic residues in its uncomplexed form [39]. Naa40 is also one of the most selective Naas since it acetylates only the Serine of the N-terminal of histones H4 and H2A. The bacterial Rims also have a narrow specificity and acetylate only ribosomal proteins (S2 Table). Naa80 is sharing the lowest BC scores with the other NATs. In addition of having a substrate-binding site wider than the other NATs, it also has a restricted substrate specificity towards the N-terminus of actin.

### 2.3 Flexibility pattern of the GNAT fold

**Fig. 5:**
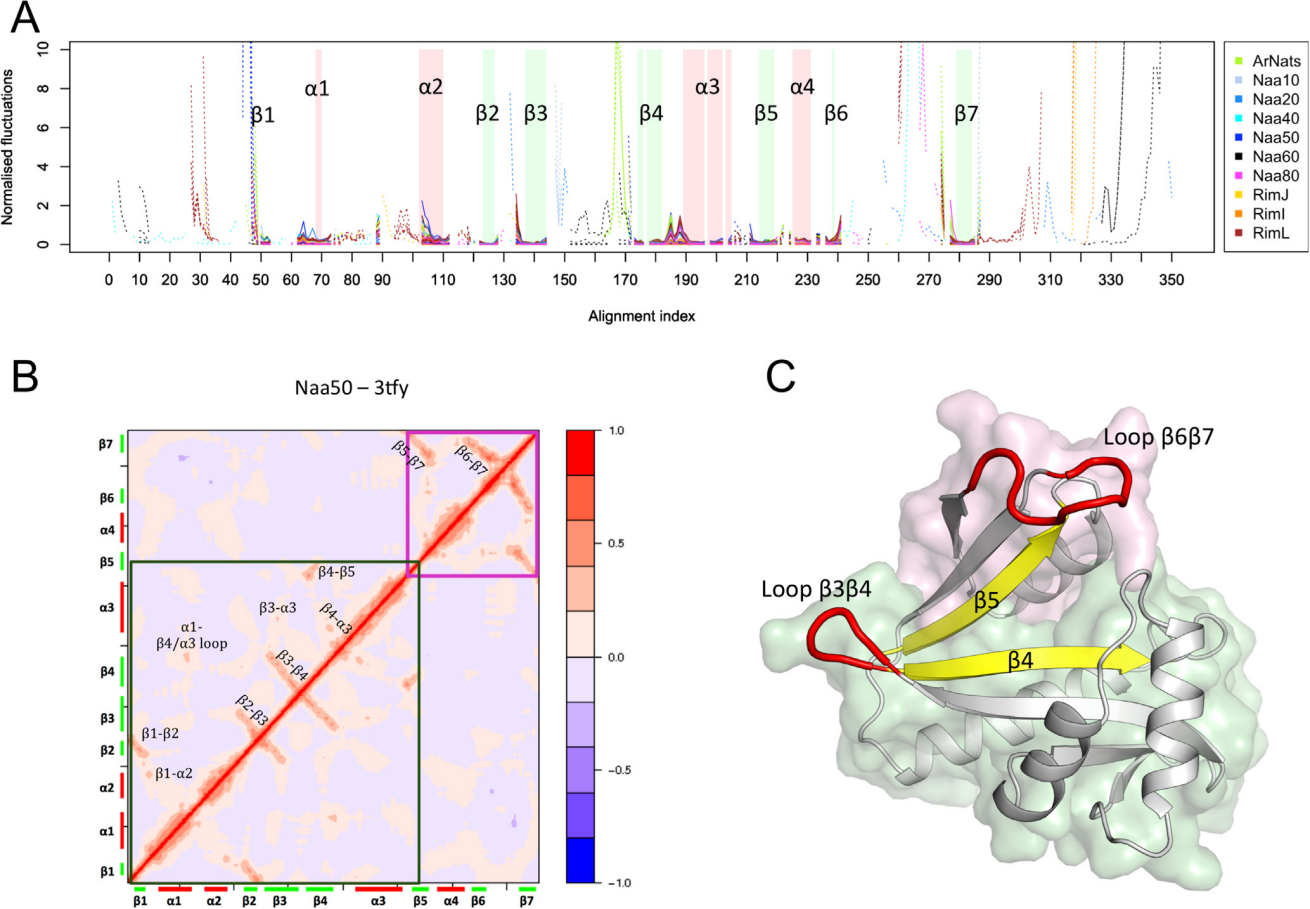
Normalized fluctuations and correlations. (A) Aligned normalized fluctuations for all NAT structures calculated with WEBnm@ [74]. For clarity one color is used for structures belonging to the same group. The fluctuations depicted in plain lines are for positions where all structures align, and dotted lines for the other positions. The secondary structures are well aligned (see also Fig. 2) and are shown here using green bars for β-strands and red bars for helices. (B) The correlation map of Naa50 (PDB ID: 3TFY) shows a correlation patterns similar to other NATs (shown in Figure S3). Long-range correlations are found within two blocks highlighted within the green and pink frames. For all NATs the highest correlations are found within these blocks and not in-between. (C) Schematic representation of the two domains (green and pink surface) revealed by the correlations. The protein is represented with a cartoon representation (here Naa50, PDB ID: 3TFY). Regions with highest fluctuations are shown in red and those with lowest fluctuations in yellow. (For interpretation of the references to color in this figure legend, the reader is referred to the web version of this article.)

Similarity in fold or topology is generally associated with similarity in flexibility and dynamics [25], [40]. We here intend to characterize the flexibility intrinsic to the topology of the GNAT fold and revealed by the high BC scores. For each structure we compute the normalised fluctuations for each amino acid and the cross correlations between pairs of amino acids, as described in Section 4 (Fig. 5A). The latter reveals how local motions are coupled across different regions of the fold. We then compare the results between NATs to reveal the flexibility patterns intrinsic to the common fold.

#### 2.3.1 Effect of Ac-CoA and the peptide substrate on fluctuations

The structures of some proteins in the dataset contain Ac-CoA and/or a peptide substrate, while others do not. Yet, and for the sake of consistency, we need to perform all ENM computations on the same system (apo enzymes) and we need to verify if removal of partner proteins, co-factor or ligand is likely to affect our conclusions. We chose the yeast Naa10 to evaluate the effect of the position of helix α2 and of the α1α2 loop, of the cofactor and of bisubstrate on the fluctuations profiles. The bisubstrate mimics the presence of both Ac-CoA and a peptide substrate [18]. The X-ray structures of the Naa10-Naa15 complex contain either a bisubstrate inhibitor (4kvm) or only a cofactor (4kvo). The structure of the uncomplexed Naa10 form has only the cofactor (4kvx). We compute the modes and the normalized atomic fluctuations of only the catalytic domain from each of the three PDB files, in the presence and absence of AcCoA and bisubstrate for the respective structures. The co-factor is represented as 11 nodes in the ENM. The beads are positioned so that they model an atom ca. every 4 Å (see Section 4). Normalized fluctuations are shown in Fig. S2. The positions of the minima (identifying rigid regions) and maxima (identifying flexible regions) are not affected by the presence of Ac-CoA or substrate. When Ac-CoA or the bisubstrate are removed from the three structures (PDB IDs: 4KVO, 4KVX and 4KVM) the mobility of β4α3 and of the N-terminal end of helix α4 increases. Further, removing the bisubstrate from 4kvm also influences the magnitude of the fluctuations of loop β6β7 since it lies close to it. We thus note that the amplitudes of the fluctuations are affected locally (i.e. at the Ac-CoA and ligand-binding sites) but the profiles remain fairly similar with the three loops β2β3, β3β4 and β6β7 being the most flexible regions of the Naa10 structure. This is in agreement with earlier works on conservation of protein intrinsic dynamics [41]. Since we are here interested in the dynamics signature of the fold and need a consistent approach, we chose to carry all subsequent calculations on apo monomeric enzymes and without Naa15 in the case of NAT A. Concretely we modified the PDB files and only retained the cartesian coordinates of one catalytic domain for the calculations. In what follows we will not investigate differences in flexibility regions involved in complex formation, or cofactor and ligand binding. This has been the subject of other computational works using an all-atoms force field that provides a better resolution for that purpose [21], [22].

#### 2.3.2 The V-shaped β-strands characteristic of the GNAT fold form a rigid core and is a hinge of movements described by the low-frequency modes

**Fig. 6:**
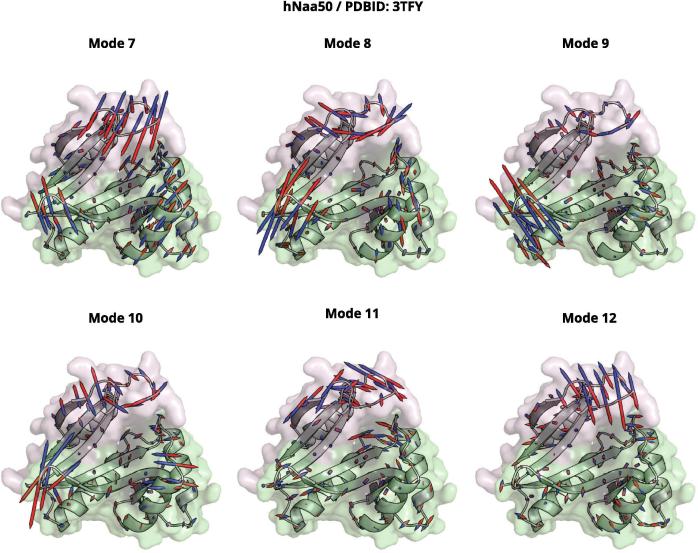
Vector field of the low frequency normal modes. Visualization of the six lowest frequency normal modes of Naa50 (PDB ID: 3TFY). The protein is represented with a cartoon representation and the surface of the two subdomains is displayed in either green or pink. The normal mode vectors are represented by arrows indicating the directions of motion. Red and blue colors depict positive and negative directions and are chosen arbitrarily. (For interpretation of the references to color in this figure legend, the reader is referred to the web version of this article.)

The normalized fluctuations are plotted on Fig. 5A. Fig. 6 shows the displacement vectors associated with the six lowest-frequency modes for Naa50 (Fig. S4 for other human Naas). We observe that the β-strands are the most rigid elements in all structures with β4 and β5 strands having the lowest fluctuations. Interestingly these two strands carry most of the catalytic amino acids (Fig. 2B) and our observation matches earlier reports of catalytic residues being positioned at particularly rigid points in protein structures [24], [25], [43], [44], [45]. Furthermore, β4 and β5 are not assembling into a sheet along their whole length despite their proximity. Instead, they form a “V shape” splitting the seven-stranded beta-sheet and creating a crevice where Ac-CoA and peptide substrate meet (Fig. 1).

In the region of the helices α1 and α2, we notice a similar pattern of flexibility between all the structures where the loop α1α2 and the helix α2 fluctuate more. Molecular dynamics simulations of the human Naa50 and Naa10 have shown that the flexibility of helix α2 is decreased in the presence of a substrate [21]. This region is also involved in the complex formation with the subunit Naa15 [22]. We observed high fluctuations for long unfolded N- and C-terminal ends. Besides those regions where fluctuations cannot be calculated reliably, the highest fluctuations are observed for the β3β4 and β6β7 loops. Two tyrosines located at positions 234 and 235 of the alignment and conserved across several groups (not in RimL, Naa40 and Naa80), are located on loop β6β7. They are known to be involved in substrate binding and form hydrogen bonds with the substrate [21]. Note that they are numbered Y138 and Y139 in the X-ray structure of Naa50, but Y135 and Y136 if the sequence is numbered from 1 as on Fig. 2.

#### 2.3.3 Structural differences in the N-terminal-α2 region leading to difference in dynamics between NATs

The GNAT fold has a region of high variability from the N-terminal to the α2 helix [7] (Fig. 2C and 4B). As noted earlier the helices α1 and α2 are longer in Naa40, RimJ and RimL than in the other NATs from group 1. RimJ and L have 1.5 additional turns in each of the two helices compared to structures clustered in group 1, while the two extra turns of helix α1 in Naa40 brings its C-terminal end over the active site at a location overlapping with that of the β6β7 hairpin loop in the other NATs. As a result, β6β7 is protruding further away from the protein core than in the other NATs and shows very large fluctuations as calculated from the modes (Fig. 2C and Fig. 5A). Furthermore, Naa40 has an extra N-terminal helix α0, the movements of which are correlated with strands β3 and β4 as well as with loop α3β5 (Cf. Fig. S3).

#### 2.3.4 The GNAT fold is divided in two dynamical domains on either side of the β5 strand

We calculated the correlations for each of the representative structures. The correlation map for Naa50 is shown as a heatmap in Fig. 5B. The maps of the other NATs share the same pattern consisting of two blocks with relatively little correlations between them (Fig. S3) indicating that the proteins contain two dynamical domains [46]. The boundary between the two coincides with the V-shape split between β4 and β5; the first domain starts at strand β1 and ends before strand β5, and the second domain starts at strand β5 and ends with strand β7. Within each domain, pairs of neighbouring β-strands are strongly correlated, as expected for beta-strands involved in the same sheet [47], but to a lesser extent for β4-β5. This is explained by the distance between β4 and β5 and the split in the fold at the β4-β5 interface. Correlations between strands and helices are weaker in general and happen through extremities of helices only. This is in agreement with what we observed for enzymes with the TIM barrel fold [25]. Correlations between domains highlighted on Fig. 5B and 5C are weak. The values of the correlations within each of those domains are below 0.2 in absolute value and at most up to 0.4 between, on the one hand, the segment containing α1, α2 and the loop between them, and β6β7 on the other hand.

Overall our calculations indicate two regions, one on either side of the split between β4 and β5, that move with respect to each other in the low frequency modes.

### 2.4 Influence of low-frequency modes on the ligand binding sites

**Fig. 7:**
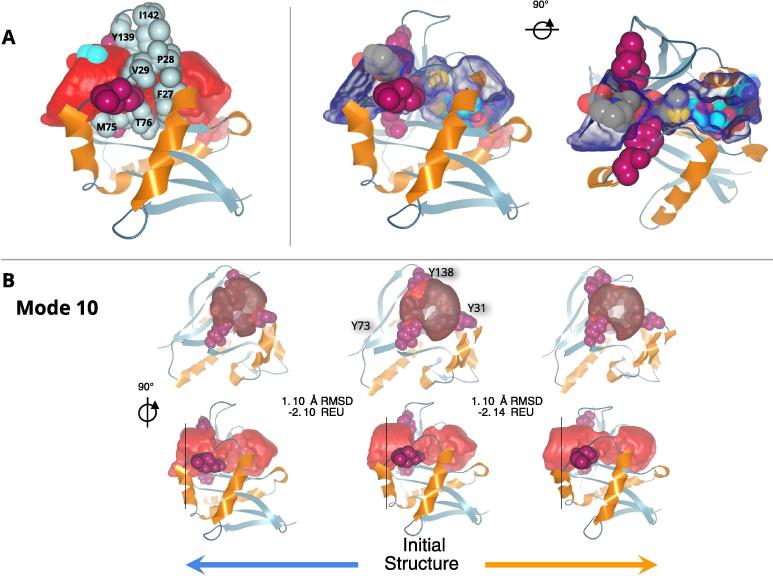
Naa50 (PDB ID: 3TFY) tunnel in the native structure and under normal mode 10. (A) Right-hand panel: native structure from the side and in cartoon representation with light blue β-strands and orange α-helices. The tunnel calculated by CAVER Analyst (see Section 4) is visualized as a red surface and the residues around the constriction are shown in light gray balls with their respective label. We show in violet three amino acids Y138, Y31, and Y73 that line the tunnel. The atoms of the ligand and cofactor are shown on top-right and bottom-left sides of the tunnel, in cyan and red respectively. Middle and left-hand panel: side and top views of the protein, respectively. The tunnel is shown as a blue transparent surface. The ligand and cofactor are shown through the cavity; carbon atoms are represented in gray for the ligand and cyan for the cofactor. (B) The initial X-ray structure is shown in the middle and the right and left structures are models generated along mode 10 in the positive (following the red arrows) and negative directions respectively (following the blue arrows). The red surface represents the tunnel. Images at the upper row are front views of the protein and its tunnel clipped by the plane indicated on the lower-row by a black vertical line. The cavity constriction is noticeable with the blur effect inside the cavity surface on the front view. The RMSD values with respect to the initial structure are given under each of the models generated along the mode. We also provide the Rosetta score in Rosetta Energy Units (REU). (For interpretation of the references to color in this figure legend, the reader is referred to the web version of this article.)

**Fig. 8:**
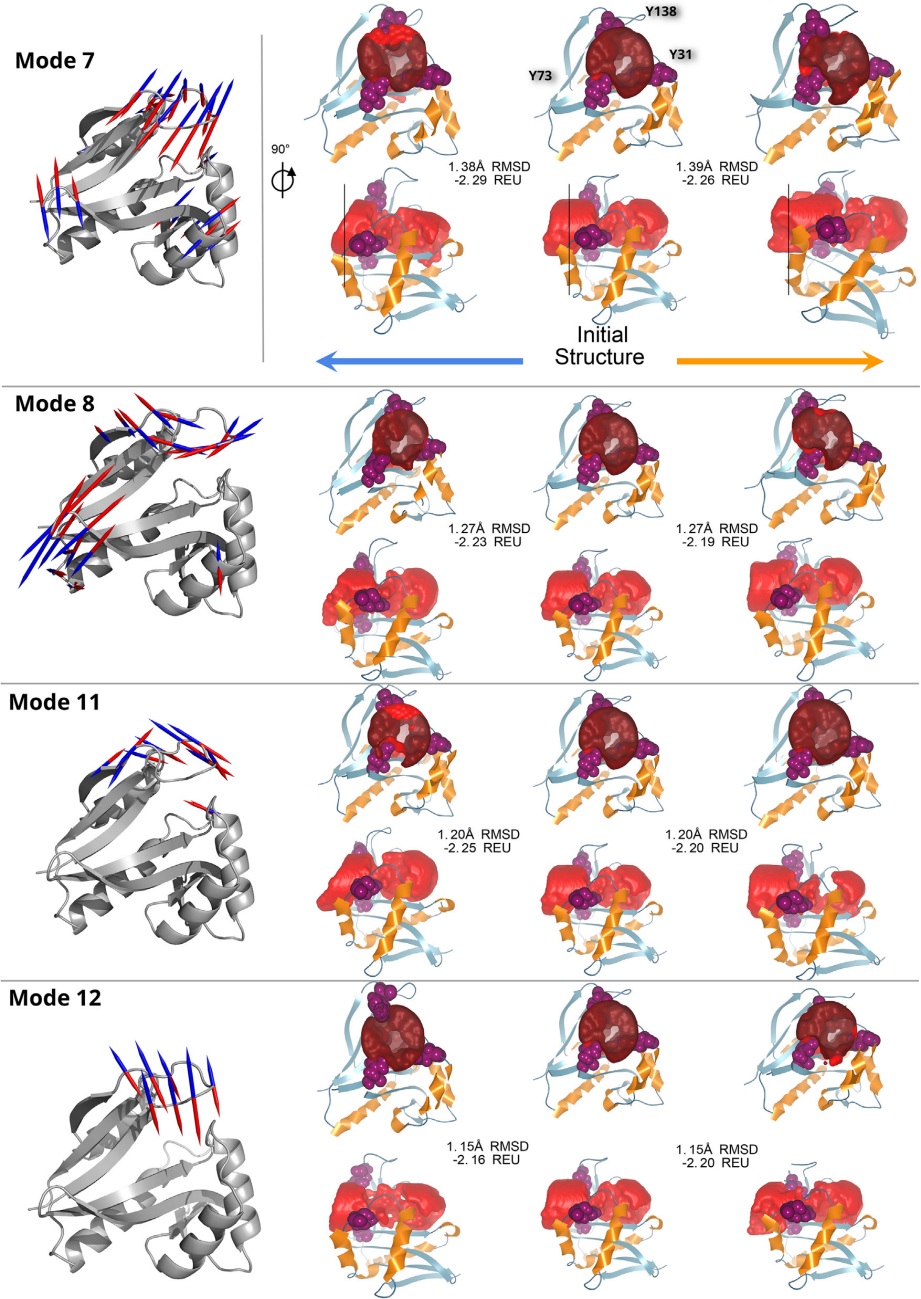
Influence of the flexibility of the loop β6β7 on the ligand cavity constriction on Naa50 (PDB ID: 3TFY). On the left-hand side, the protein structure is represented in grey cartoons and the selected normal mode vectors are represented by arrows indicating the positive and negative directions of motion (in red and blue respectively). For the sake of clarity only the largest displacements are shown (Cf. Fig. 6). On the right-hand side, the structure is represented in cartoons with light blue β-strands and orange α-helices. The middle structure depicts the initial X-ray structure and the right and left structures, the corresponding mode deformations in positive (following the red arrows) and negative directions respectively (i.e. following the blue arrows). The red surface represents the tunnel calculated by CAVER Analyst (see Section 4). On each horizontal panel, corresponding to each selected normal mode, the upper row represents a front view of the protein and its cavity clipped by the plane indicated on the lower-row side view by a black vertical line. The cavity constriction is noticeable with the blur effect inside the cavity surface on the front view. (For interpretation of the references to color in this figure legend, the reader is referred to the web version of this article.)

Since the ligand binding site is located at the interface between the two domains it is likely affected by the movement of the domains. Moreover, given the mobility of the β6β7 loop (Cf. Fig. 6) and its position with respect to Ac-CoA and the substrate binding site, its movements might also influence the fairly narrow tunnel in which substrate and Ac-CoA meet (Cf. Fig. 1). In order to determine the contributions of loop motions to the accessibility of the active site, we use CAVER to compute the walls of the tunnel on several Naa structures and then evaluate the changes to the tunnel when the conformation is modified along the individual low frequency normal modes (see Section 4). As these calculations and their analysis are tedious, we restricted the study to Naa10, Naa20, Naa40, Naa50, Naa60 and Naa80. For each protein the calculation of the tunnel is performed on the native structure and on two additional conformations generated along the displacement vectors of the normal modes (See Section 4). We show the results for Naa50 on Fig. 7, Fig. 8 (see Fig. S5 for other NATs).

The tunnel calculated for the X-ray structure of Naa50 (PDB ID: 3tfy) is shown on Fig. 7A. The walls of the tunnel are defined by the position of amino acids belonging to several structural elements. The amino acids lining the tunnel are: the side chains of 5 amino acids from β4 (Y73, I74, M75, T76, L77), 4 amino acids from β5 (L111, H112, V113, Q114), 5 amino acids from the loops α1α2 (F27, P28, V29, S30, Y31) and 5 residues from β6β7 (K135, Y138, Y139, R140, I142). We also observe sidechains from the α1 (F27), α2 (N32, K34, F35) and α4 (Y124) helices delimiting the tunnel, as well as β3 with R62. The tunnel in the initial structure shows a clear constriction close to the position of the active site where substrate and co-factor meet. Actually, one can see on Fig. 7 that the side chain of the N-terminal Met is beyond the constriction. It also shows that amino acids forming this constriction in the tunnel belong to the loops α1α2 and β6β7. So the tunnel walls in Naa50 are formed primarily by 5 structural elements on the ligand side: β4 and β5, which are not mobile (Cf Fig. 5), and α1α2, α2 and β6β7 which are all mobile though with a varied range of amplitudes. In the other NATs, the tunnel is also formed by these structure elements (with the addition of α1 for Naa40 where the helix is longer and covers the active site), and β3 for Naa80 where the cavity forms a cleft, exposing the core β-strands of the protein. In Naa20 the tunnel seems overall narrower. The constriction formed by the α1α2 and β6β7 loops in Naa50 is also present in other NATs, except for Naa80 where β6β7 is very long (Fig. S5). In Naa40 the constriction extends longer than in the other NATs (Cf Fig. S5).

**Fig. 9:**
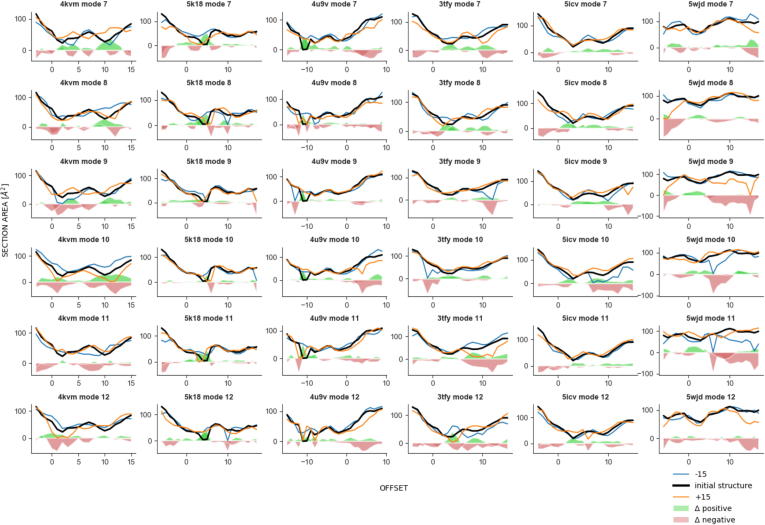
Cross section areas of the ligand binding site in human NATs. The cross section areas are plotted against the offset. The offset is the position of the cross-section along the cavity centerline. The areas are plotted for the native structures (black lines) and models generated along the six lowest frequency normal modes in either negative or positive directions (blue and orange lines). The data is organized so that each column corresponds to a structure, namely from the left-hand side: Naa10 (PDB ID: 4KVM), Naa20 (PDB ID: 5 K18), Naa40 (PDB ID: 4U9V), Naa60 (PDB ID: 5ICV), and Naa80 (PDB ID: 5WJD). Each row corresponds to a normal mode, from 7 to 12, from top to bottom. The offset origin is set at the section crossing the ligand binding site. The red and green areas represent the computed difference between the black lines and the orange or green lines, respectively. In other words, they represent the gain (in red) or loss (in green) of cross-section area caused by modifying the enzyme structure along the modes in the positive (red) or negative (green) direction. (For interpretation of the references to color in this figure legend, the reader is referred to the web version of this article.)

Fig. 9 represents the tunnel cross-section area along the tunnel (black line) for each of the human Naas. It also shows how the cross-section is modified following each vibrational mode in one direction (blue line) or the other (orange line). Following the black lines one can clearly see for all proteins except Naa80 the position of at least one constriction between the opening with an area at about 100 Å^2^ or more on the ligand-binding sites (left-hand side of each plot, negative offset) and the AcCoA binding site on the right-hand side. Besides this constriction, and despite generally resembling shapes, the section area profiles differ from one Naa to another. Yet, they all have in common that most low frequency modes modify the cross-section area at the constriction site (see the green and red areas on the plots of Fig. 9). They are modes 7, 9, 10, 12 for Naa10; 7, 8, 11, 12 for Naa20; 7–12 for Naa40; 7, 8, 10, 12 for Naa50; 8, 9, 10, 12 for Naa60. The tunnel in Naa80, though modified by most modes, does not really have a narrow constriction.

Helix α2 is somewhat mobile according to the normalized fluctuations shown on Fig. 5. Its movements towards or away from the β4 and β5 strands influence the size of the tunnel opening in the same manner in Naa10, Naa20, Naa50 and Naa60. This is particularly visible for example for mode 8 of Naa50 (Fig. 8) or modes 7 and 12 of Naa20 (Fig. S5). However the movements of α2 have a different effect in Naa40. For instance, in modes 10 and 11, despite the fact that the helix moves away from the structure core, the tunnel mid-section shrinks towards the active site (Fig. S5 and Fig. 9). This can be explained by a long α1 helix and a long α1α2 loop, placing the loop higher above the active site than in the other structures and covering it from above. It thus constricts the tunnel in a different manner than it does for the other NATs.

The β6β7 loop is one of the most mobile elements according to our calculations. One can see the effect of its displacements on the ligand binding site on Fig. 7B which illustrate the tunnel changes in mode 10. Although mode 10 does not involve large displacements of the β6β7 loop compared to the other modes (Cf. Fig. 6), it still modifies the contours of the tunnel; in particular at the opening where small displacements of Y138 modify the cross-section area (see Fig. 9). The modes 7, 8, and 12 involve larger displacements of β6β7 and are shown on Fig. 8. They involve changes of the cross-section area at the position of the constriction (Fig. 9). We chose to leave mode 9 out of Fig. 8 for the sake of clarity and because the associated movements do not have a visible effect on the tunnel. Regarding other structures, the behavior of β6β7 residues is conserved in mediating the constriction size at this location in the tunnel. The effect is clearly visible for Naa10 (modes 7, 9, 10, and 12), as well as for Naa20 (modes 7, 8, and 9) for instance (see Fig. 9).

The β4α3 loop is on the side of the AcCoA binding site and does not influence the ligand entrance site but has effects restricted to the cofactor binding site. In Naa50, the movements of the β4α3 loop (R84, and A81) and of the N-terminal end (S119, F123) of α4 are important along modes 11 and 12. These movements lead to large changes of the cross-section area, especially so for mode 11 (Fig. 9). For the other human NATs, the β4α3 also modifies the right end of the cavity, for instance for Naa10 in modes 9 and 10, or Naa40 with modes 7, 8, and 10, or Naa60 with modes 9 and 10 (Fig. 9).

The displacements of the 4 amino acids long β3β4 loop in Naa50 are large in modes 7 to 10, almost as large as that of loop β6β7 which counts 11 amino acids and is fairly structured. In Naa10, large motions of this loop are observed for modes 8, 9, 11, and 12, or in Naa20, for modes 9 and 10. We cannot see that the movements of the β3β4 loop have a direct effect on the ligand binding site even though given the magnitude of the displacements in the low-frequency modes we suspect that they have a functional role.

The results described above indicate that the loops α1α2 and β6β7 have the ability to shape the access route to the cofactor, each having different effect on the tunnel, at different positions along its axis (see side views on Figures 7 and 8, and S5). They appear as dynamic steric regulators of the entrance to the catalytic site. The dynamics of β4α3 has effects most likely restricted to the AcCoA binding site.

In total, most low frequency modes of Naa50 and of the other NATs modify the shape and cross-section area of their ligand binding site, so we are confident that the dynamics of the GNAT fold affects the size and shape of the binding site in a way that might regulate ligand uptake and release.

In principle vibrational normal modes inform about movement directions and amplitudes. In our case, we use a cost-effective coarse-grain model (Cα only) to be able to perform the same calculations on many structures. With this coarse model we cannot predict the absolute amplitude of the modes. We assign it semi-arbitrarily when we generate the conformations shown on Fig. 7, Fig. 8 where we make a conservative estimation to avoid unrealistic local deformations and so that the generated conformations remain close to the X-ray structure (at most 1.38 Å RMSD for Naa50, and below 2 Å for all proteins, Cf. Section 4). For this reason we cannot predict the exact changes in the size of the ligand binding site. Moreover, it is expected that the amplitude of the movements can be modulated by interactions between the catalytic domains and either the co-factor, ligand or other domains as discussed in Section 2.3.

## 3 Discussion

The variety of NATs enables the selective acetylation of a diversity of N-termini of proteins at different localizations in the cell. The GNAT fold shared among all NATs and with other acetyltransferases offers a common scaffold to perform the catalytic activity and fine-tune ligand selectivity. One particular feature of the GNAT fold in the NATs is a very tight access to the catalytic site, which is shielded by the β6β7 hairpin loop and the α1-α2 region forming a tunnel together. Using ENM-NMA we have compared the intrinsic dynamics of the GNAT fold in all available structures of NATs covering all domains of Life. We describe the dynamic pattern intrinsic to the fold and common to all NATs catalytic domains. It entails movements that subtly affect the size and shape of the ligand binding sites and potentially regulate the access of the ligand to the cofactor and catalytic site, through a narrow tunnel. For the sake of consistency, our study is restricted to the catalytic domains. This approximation is validated by earlier works showing that slow intrinsic dynamics is encrypted in protein folds and that direction of movements and fluctuation profiles of a given domain in its apo form will not be significantly perturbed by protein–ligand or protein–protein interaction [25], [40], [48]. Also, we earlier reported the fluctuations of Naa10 in the Naa10-Naa15 complex using molecular dynamics simulations [42]. We found then that the loops β3-β4 and β6-β7 were the most flexible elements of the fold, similar to what we report in this study.

The intrinsic dynamics of the GNAT fold is characterized by a rigid β-sheet core, not unexpectedly as β-sheet regions are known to be rigid [25] though their deformation modes show a high degree of correlation and can transmit mechanistic signals between distal sites [47]. Of all the strands, β4 and β5 are the least flexible but they are not tightly packed along their whole length and form a V-shape split. Less expected than the rigidity of the β-sheet region is that the split defines two regions that behave as independent rigid bodies in the low-frequency normal modes, dividing not only the β-sheet region but the GNAT fold in two subdomains. The region between the C-terminal end of α3 and the N-terminal end of β5 thus contains hinge residues that are pivots in the larger motions of the protein. Interestingly the pathological Naa10 p.V107F mutation at the N-terminal end of β5 causes a 95% reduction of the catalytic activity compared to Naa10 WT [49]. In this study, Popp et al*.* built a homology model of the mutant V107F and observed a disruption of hydrophobic contacts with the Met98 found on helix α3 of the human Naa10. Seen in light of our results, we would suggest that the mutation perturbs the packing density at hinges and consequently affects function through a modification of the intrinsic structural flexibility [50].

The β4 and β5 strands carry residues involved in the proton wire essential for the transfer of the acetyl. The rigidity of the catalytic core in the GNAT fold is in agreement with case-studies of enzyme dynamics where residues involved in catalysis are found to be placed at rigid conserved positions of the fold [25], [40], [44] while substrate-binding residues tend to be in more flexible regions [40], [51], [52]. The catalytic site is thus at the crossroads of the two domains and flanked by the α1α2 and β6β7 loops. Together with β3β4, they are the most mobile regions in NATs, irrespective of their length. Several of the residues important for substrate binding are carried by the α1α2 and β6β7 loops [21].

Besides the dynamics patterns described above and the similarities in flexibility between NATs (Cf. high BC scores), there are structural differences among the NATs which naturally are reflected in their dynamics. For example, there are differences in the regions that span from the N-terminal to α2 and in the region of the β6-β7 loop and those structural differences might affect ligand binding. In particular the calculation of the tunnel in Naa20 reveals a strong constriction closer to the active site and an overall seemingly narrower tunnel. The α1α2 loop in Naa20 is more tilted towards the active site and helix α1. This difference causes the residue L23, to be oriented towards the active site and α4 helix and forming the longer constriction. This particular orientation could be explained by the Thr24 residue, replaced by a proline in Naa10, and shifting the α1α2 loop towards the end of the tunnel. In Naa10, the leucine is known to be involved in substrate binding, whereas this role is transferred further along the loop sequence in Naa20 [53]. In general, the amplitude of the displacements of those structural elements will vary with their lengths and initial position with respect to the protein core. Naa40, RimL and RimJ have longer α1 and α2 helices and a longer α1α2 loop, which results in slight changes of the active site shape. This was also captured by the BC score, which quantifies similarities of intrinsic dynamics between the aligned core regions of proteins. We highlighted flexibility dissimilarities between Naa40, the bacterial RimL and RimJ in one hand, and the rest of the NATs in the other hand. Naa80 clusters on its own. As far as we are aware of, Naa40 is the only known NAT with a different position of the substrate in the active site. As shown by Magin et al., all the substrates in other structures have their 2nd and 3rd residues sitting close to the α1 and α2 helices, while the α2 helix in Naa40 obstructs this region shifting the substrate towards the β5 and β7 strands [20]. The bacterial RimL is only active as a homodimer unlike the other NATs, which tend to be active as monomers complexed with auxiliary subunits forming heterodimers or heterotrimers (S2 Table). The β6β7 loop is part of the RimL dimerization interface. Helix α2 is tilted away from β4 yielding a larger opening of the cavity compared to the other NATs. The longer elements in Naa40, RimJ and RimL thus illustrate how secondary structure elements lining the binding site affect its size, shape, and accessibility. In the case of Naa80, the opening to the binding site is wider than in other NATs and this is thought to play a role in its specificity for the acidic actin N-termini [37].

As described higher up in this section, the low-frequency modes in NATs displace the two sub-domains of the GNAT fold with respect to one another and result in large movements of the β6β7 hairpin loop, and of the α1α2 and β3β4 loops. The impact of these motions on the shape and size of the entrance of the substrate binding site is striking even with relatively small deformations of the X-ray structure along the modes. The actual conformational changes of NATs might use a combination of the low-frequency modes and amplitudes which we cannot reliably predict with an ENM-NMA. Yet our results, including both the tunnel computations and the analysis of the changes in its shape and cross section, are a strong indication that the ligand needs movements from the loops to be able to progress in the tunnel and reach the catalytic site and the cofactor. Moreover the modulation of the amplitudes of the loop movements by additional domains might be a mechanism to regulate the access to the substrate binding site, and modulate the ligand specificity.

The effect of the S37P mutation in the C-terminal end of helix α2 in the human Naa10 is causative of the lethal Ogden syndrome [4]. The mutation impairs the catalytic activity and the formation of the NatA complex, inducing a reduction of NatA-mediated N-terminal acetylation and affecting cell proliferation [4]. Using molecular dynamics simulations on the model of the human NatA complex, we have earlier shown that this mutation decreased the fluctuations of the α1α2 loop and of the α1 helix. The fact that it impairs the catalytic activity is an indication that flexibility of regions remote from the catalytic site are important for ligand and/or cofactor binding sites. It supports our observations that the dynamics of the overall proteins is important for ligand binding and/or catalysis. The α1α2 and β6β7 loops might be the actual effectors by shaping the tunnel, but their movements are part of concerted subtle structural changes in the fold.

Kurkcuoglu et al. studied a set of ten enzymes for which the active site can be in both an opened and closed form. They showed the role of global motions of functional loops in assisting the binding and positioning of substrates, and that these motions were energetically accessible to the enzymes in the absence of substrates [54]. In the case of the NATs, we cannot exclude that the β6β7 loop opens the tunnel entrance or bottleneck more than we observe in the conformations we generate along the modes.

It is worth noting that NATs can be inhibited by so-called bisubstrate inhibitors consisting of a short polypeptide covalently bound to the Ac-CoA [6], [18], [55], [56]. The X-ray structure of the human NatF bound to bisubstrate CoA-Ac-MKAVQAD-NH_2_ (CoA-Ac-MKAV_7_) shows that the inhibitor is placed in the Ac-CoA and substrate binding site with the β6β7 hairpin loop hanging over the top of it [55]. It raises the question of how the bisubstrate accesses both the ligand and Ac-CoA binding sites and, as a consequence, suggests that (i) the tunnel opens up enough to let the long bisubstrate thread through the tunnel or (ii) that the β6β7 loop opens as a lid to let the bisubstrates bind from above.

Another interesting question is that of the acetylation of internal lysines by NATs. Lysine acetyltransferases (KATs) perform protein acetylation to lysine side chains while NATs acetylate N-terminii. There is no strand equivalent to β6 in KATs; the NATs’ strand-loop-strand motif formed by β6 and β7 is replaced by a loop-strand motif which allows easier access to the ligand binding site [17], [18], [20]. The α1α2 and β6β7 loops have been proposed to prevent the access of internal lysines to the catalytic site and as a consequence prevent their acetylation by NATs [17], [20]. However, there have been reports of acetylation of internal lysines by NATs [19], [57], [58], [59], [60], [61], [62], [63] and we suggest that this could be facilitated by the dynamics of the loops. The human Naa10 has been shown to acetylate internal lysines of various proteins [60], [62], [63], [64] and the auto-acetylation on its K136 found on the β6β7 loop could be the reason of its shift of substrate specificity towards internal lysine [65]. Movements of the loop, or stabilization in an opened position, might be enabled or triggered by either a particular substrate or experimental conditions.

In summary, we propose that the dynamics of the two domains and the high mobility of the β6β7 loop give the ligand binding site a flexibility that is important for its substrate binding and selectivity. Our calculations show that fairly small rigid-body displacements of the β6β7 loop modify the accessibility to the active site and the Ac-CoA. Our work fills a gap in the understanding of the versatility and broad substrate specificity of the NATs enzymes [4], [21]. Our results are relevant for those seeking to design inhibitors of NATs involved in cancer, Huntington’s disease or other pathologies. Further investigations are needed to experimentally evaluate the extent of the influence of the loop mobility on NATs activity and substrate specificity. This could be done by mutagenesis experiments where selected amino acids in hinge regions could be replaced by glycine or proline to increase or reduce loop mobility. Such an approach would present the advantage of not affecting the structure and stability of the β-hairpin itself [66], [67].

## 4 Methods

### 4.1 Dataset preparation

NATs do not constitute a specific group in fold databases CATH [68] and SCOP [69], [70]. To generate our dataset of structure we thus collected structures from PDBe [71] using two filters: the annotation of GNAT domain from PROSITE [72] (PROSITE code: PS51186) and “N-terminal protein amino acid acetylation” as a biological process. We collected more than 160 structures that we filtered down to 45 structures being annotated as N-alpha acetyltransferases or N-terminal acetyltransferases. All are listed in Tables 1 and S1. From these we excluded eleven structures for which the X-ray structure had unresolved segments within the GNAT fold. These are written in grey in Table 1 (and in Table S1).

We formed 10 functional groups: Naa10, Naa20, Naa40, Naa50, Naa60, Naa80, archaeal NATs, RimI, RimJ and RimL. These groups were formed either by considering the Enzyme Commission (EC) number, their functional annotation in scientific literature when available or the kingdom of the organism the protein is found in. All structures files were prepared for the calculations by selecting one chain in the assembly and removing the Ac-CoA or peptide substrate if present. The reference set consists in one structure, called representative, for each Uniprot code in each functional group (see PDB IDs in bold in Table 1).

### 4.2 Structural alignment

In order to compare the intrinsic dynamics of the NAT structures in our dataset we need a good structure-based alignment of their sequences [25], [26]. We thus generated a structural alignment using MUSTANG [33] which has been shown to perform very well on distant related proteins [25], [26]. The algorithm performs a progressive pairwise alignment using the position of Cα atoms. It extends the pairwise structural alignments into multiple structure alignments by recalculating a pairwise residue-residue score at each step of the extension and progresses using a guide tree. We show the pairwise RMSD between Naas structures, and how it clusters the structures together, on a heatmap plotted with the R function pheatmap [73].

### 4.3 Elastic network model and normal mode analysis (ENM-NMA)

The normal mode analysis was performed using WEBnm@ [74]. The web-tool uses an Elastic Network Model (ENM) modelling protein structures as a network of nodes, the Cα atoms, connected together by Hookean springs. We used the Calpha force field [75], [76], as implemented in the Molecular Modelling Toolkit [77]. It uses a pair potential to describe the interactions between two Cα atoms as:

$$V_{\mathrm{ij}}\left(r\right)=\frac{k_{\mathrm{ij}}}{2}{\left(\|r_{\mathrm{ij}}\left\|-\right\|r_{\mathrm{ij}}^{0}\|\right)}^{2}$$

where $r_{\mathrm{ij}}$ is the distance vector between two Cα atoms i and j in the configuration r of the protein, $r_{\mathrm{ij}}^{0}$ is then the same pairs of atoms i and j at the equilibrium conformation and $k_{\mathrm{ij}}$ is the non-uniform force constant defined by:

$$k_{\mathrm{ij}}=\left\{\begin{array}{c} ar_{\mathrm{ij}}^{0}-b,\;\;for{\;r}_{\mathrm{ij}}^{0}<d\\ {c\left(r_{\mathrm{ij}}^{0}\right)}^{-6},\;\;for{\;r}_{\mathrm{ij}}^{0}\geq d \end{array}\right)$$

with $a=8.6\times 10^{5}\;{kJmol}^{-1}\;{nm}^{-3}$; $b=2.39\times 10^{5}{\;kJ\;mol}^{-1}{\;nm}^{2}$; $c=128\;{kJ\;mol}^{-1}\;{nm}^{4}$ and d=0.4nm.

The potential energy of the network model is the sum of all the atomic configurations:

$$V\left(r\right)=\sum_{i=1}^{N}\sum_{j=i+1}^{N}V_{\mathrm{ij}}\left(r\right)$$

where N is the number of nodes in the network.

For the normal modes calculation of the holo form of *Schizosaccharomyces pombe* Naa10 we represented CoA by 11 beads placed at the positions of atoms distant of 3–4 Å, namely C, C3P, C6P, C9P, CCP, P1A, C4B, P3B, N9A, N6A and N3A.

### 4.4 Normalized fluctuations

The fluctuations $F_{i}$ give the variances of each atom position and are given by:

$$F_{i}=\sum_{m=7}^{3N}\frac{{\|{\left[d_{m}\right]}_{i}\|}^{2}}{\lambda_{m}}$$

where $d_{m}$ is the displacement vector of the atom i in mode m. $F_{i}$ is then the sum of all the squared displacements of i for all the non-trivial modes that are weighted by their eigenvalues.

### 4.5 Correlations

The matrix of correlations is calculated from the normal modes [78] which quantifies the coupling between two atoms i and j as:

$$C_{\mathrm{ij}}=\frac{\sum_{m=7}^{3N}\frac{1}{\lambda_{m}}{\left[v_{m}\right]}_{i}∙{\left[v_{m}\right]}_{j}}{{\left(F_{i}\right)}^{\frac{1}{2}}{\left(F_{j}\right)}^{\frac{1}{2}}}$$

where $v_{m}$ and $\lambda_{m}$ are the eigenvector and eigenvalue of a non-trivial mode m. $C_{\mathrm{ij}}=1$ when the motions are completely correlated and $C_{\mathrm{ij}}=-1$ when they are completely anti-correlated.

### 4.6 Bhattacharyya coefficient score

Finally, we used the Bhattacharyya coefficient (BC) score to compare the effective covariances of the common aligned cores of two structures A and B as implemented in WEBnm@ [74] and described in Fuglebakk et al. [38]:

$$BC\left(p_{a},p_{b}\right)=\frac{{\left|\tilde{A}\right|}^{\frac{1}{4}}{\left|\tilde{B}\right|}^{\frac{1}{4}}}{{\left|\frac{1}{2}\left(\tilde{A}+\tilde{B}\right)\right|}^{\frac{1}{2}}}$$

where $p_{a}$ and $p_{b}$ are the Boltzmann distributions of structures A and B, and $\tilde{A}$ and $\tilde{B}$ represent the covariance matrices of the common aligned cores of the two structures.

### 4.7 Generation of Cα and all-atoms trajectories

We generated trajectories of the Cα models along the normal modes. This was done in order to visually inspect structural changes of the NATs and to analyze the position and shapes of the ligand-binding sites for the eukaryotic NATs. The latter required that we add the sidechain and mainchain atoms to the Cα trajectories. The procedures used to generate the Cα and all-atoms trajectories are described below.

We selected the 6 first non-trivial modes from the set of normal modes of the human Naa10, Naa20, Naa40, Naa50, Naa60 and Naa80. For each mode, we generated structural models by displacing the initial Cα positions following the mode displacement vectors in either directions (positive and negative). The deformed structures were optimized by minimizing the energy of their elastic network in the normal mode space as described in Ref. [79].

The generation of the all-atoms trajectories for the human NATs was adapted from Mahajan and Sanejouand [80] and consists in 3-steps: generating conformations along the modes, minimization of the elastic network energy, and side chain reconstruction. It was carried out at six different amplitudes (from 3 to 18 and in each direction) to find the largest displacement we could apply without distorting the structure unrealistically. We first used the Molecular Modelling ToolKit (MMTK [77]) to reconstruct the main chain and side chains on each structure from the Cα trajectories (see above). We calculated the 3D transformation necessary to superimpose the initial all-atom structure onto each generated Cα model. This was done by minimizing the RMS difference between the two. The 3D transformations were not computed on the overall structure but locally using an iterative process; we used sliding windows that were three amino acids-long to compute the transformation. This transformation was then applied to the central amino acid for which the side chain is reconstructed. The process is then iterated by sliding the window along the protein sequence by one residue.

All resulting structures where relaxed using the relax protocol of pyROSETTA [81], [82] (version 2019.33+release.1e60c63beb5) and the ref2015_cst scoring function [83]. In order to keep the normal mode displacement information intact, we applied constraints on the protein backbone. Also, the pyROSETTA relax algorithm option to control the standard deviation allowed for coordinate constraints was set to 0.5 as recommended by Nivón et al. [84]. We observed that at an amplitude factor of 15 we still retained good Rosetta energy scores for the six proteins and the first 6 modes (modes 7–12). We thus selected 15 which, for the sake of consistency, was applied to all 6 proteins. The resulting RMSDs are all below 2 Å. RMSD calculations are performed on the structurally aligned Cα atoms (see Structural alignment section). The Rosetta score (in Rosetta Energy Units, REU) for Naa50 and modes 7, 8, 10, 11 and 12 is shown on Fig. 7, Fig. 8 (and on Fig. S5 for Naa10, Naa20, Naa40, Naa50, Naa80), as well as the RMSD between Cα of the initial structure and each selected conformation. The energy scores were normalized with respect to the size of the proteins and are given per residue.

The generated protein conformations were thus generated from displacing the initial structure following each mode separately and up to a carefully chosen extent so that the structure remains close to the initial X-ray structure.

### 4.8 Calculation and visualisation of ligand-binding site tunnels

The analysis was performed using CAVER Analyst [85]. We first selected the Cα atoms from three amino acids: Y31, Y73, and Y138 in Naa50. They were chosen because they are lining the opening of the tunnel in the X-ray structure. For other proteins, we chose amino acids at equivalent positions with respect to the ligand binding site (all listed on Fig. 7, Fig. 8 and S5). In 3D space, these three atoms define a plane perpendicular to the tunnel axis. Then a set of intersecting spheres, with a radius of 1 Å, is placed on a line perpendicular to this plane and with a length of 30 Å. Using this geometrical structure as a base, we compute and extrude the cavity surface up to 6 Å in width for each frame and using the algorithm described in [85]. We do not use the extension of the algorithm proposed by Jurcik et al. [86] as it was developed for the detection of deeply buried voids inside proteins. While in our case the cavities in NATs are not fully surrounded by amino acids and are closer to the protein surface. Here, the protein surface is calculated using a surface probe of 15 Å.

## CRediT authorship contribution statement

**Angèle Abboud:** Conceptualization, Data curation, Formal analysis, Investigation, Methodology, Visualization, Writing - original draft, Writing - review & editing. **Pierre Bédoucha:** Formal analysis, Investigation, Methodology, Software, Visualization, Writing - original draft, Writing - review & editing. **Jan Byška:** Methodology, Software, Supervision, Writing - review & editing. **Thomas Arnesen:** Conceptualization, Supervision, Writing - review & editing. **Nathalie Reuter:** Conceptualization, Funding acquisition, Methodology, Project administration, Supervision, Validation, Writing - original draft, Writing - review & editing.
