# Supplementary material for: Dynamics-function relationship in the catalytic domains of N-terminal acetyltransferases
Source: Comput Struct Biotechnol J. 2020 Mar 3;18:532–47. doi: 10.1016/j.csbj.2020.02.017 (PMC7078549; doi:10.1016/j.csbj.2020.02.017)
Supplement: Supplementary data 1 [file mmc1.docx]

Dynamics-function relationship in the

catalytic domains of N-terminal acetyltransferases

Angèle ABBOUD, Pierre BÉDOUCHA, Jan BYŠKA, Thomas ARNESEN and Nathalie REUTER^*^

# Supporting Information

**S1 Table.** **Structure dataset.**

| **Group** | **PDB ID** | **Chain** | **Oligomeric state** | **Co-factor** | **Ligand** |
| --- | --- | --- | --- | --- | --- |
| **ArNat** | 2x7b | A | Monomeric | CoA | - |
|  | 4lx9 | A | Monomeric | Ac-CoA | - |
|  | 4pv6 | A | Monomeric | Ac-CoA | - |
|  | 4r3k | A | Monomeric | CoA | - |
|  | 4r3l | A | Monomeric | CoA | Peptide |
|  | 5c88 | A | Monomeric | CoA | - |
| **Naa10** | 4hnw | B | Complexed with Naa15 | - | - |
|  | 4hnx | B | Complexed with Naa15 | - | - |
|  | 4hny | B | Complexed with Naa15 | - | - |
|  | 4kvm | E | Complexed with Naa15 | Bi-Substrate^[[1]](#endnote-1)^ | Bi-Substratei |
|  | 4kvo | E | Complexed with Naa15 | Ac-CoA | - |
|  | 4kvx | B | Monomeric | Ac-CoA | - |
|  | 4xnh | B | Complexed with Naa15, Naa50 | Ac-CoA | - |
|  | 4xpd | B | Complexed with Naa15, Naa50 | Ac-CoA | - |
|  | 4y49 | B | Complexed with Naa15, Naa50 | Ac-CoA | Peptide |
| **Naa20** | 5k04 | B | Complexed with Naa25 | CoA | - |
|  | 5k18 | B | Complexed with Naa25 | Bi-Substratei | Bi-substratei |
| **Naa40** | 4u9v | A | Monomeric | Ac-CoA | - |
|  | 4u9w | B | Monomeric | CoA | Substrate |
|  | 4ua3 | A | Monomeric | CoA | Peptide |
| **Naa50** | 2ob0 | A | Monomeric | Ac-CoA | - |
|  | 2psw | A | Monomeric | CoA | - |
|  | 3tfy | C | Monomeric | CoA | Peptide |
|  | 4x5k | A | Monomeric | CoA | Peptide |
| **Naa60** | 5hgz | A | Monomeric | Ac-CoA | - |
|  | 5hh0 | A | Homo-2-mer | CoA | - |
|  | 5hh1 | A | Homo-2-mer | CoA | - |
|  | 5icv | B | Monomeric | Bi-Substratei | Bi-Substratei |
|  | 5icw | A | Homo-2-mer | CoA | - |
| **Naa80** | 5wjd | A | Monomeric | Ac-CoA | - |
|  | 5wje | A | Monomeric | Bi-Substratei | Bi-Substratei |
| **RimJ** | 3igr | A | Homo-2-mer | - | - |
| **RimI** | 2cnm | A | Monomeric | Bi-Substratei | Bi-Substratei |
|  | 2cns | A | Monomeric | Ac-CoA | - |
|  | 2cnt | A | Monomeric | CoA | - |
|  | 5isv | A | Monomeric | - | - |
| **RimL** | 1s7f | A | Homo-2-mer | - | - |
|  | 1s7k | A | Homo-2-mer | - | - |
|  | 1s7l | A | Homo-2-mer | CoA | - |
|  | 1s7n | A | Homo-2-mer | CoA | - |
|  | 1z9u | B | Homo-2-mer | - | - |
|  | 2z0z | A | Homo-2-mer | - | - |
|  | 2z10 | A | Homo-2-mer | - | - |
|  | 2z11 | A | Homo-2-mer | - | - |
|  | 2zxv | A | Homo-2-mer | - | - |

Bi-substrate refers to a compound where a peptide and CoA are covalently bound [1]

**S2 Table.** **Ligand specificity of NAT enzymes included in the dataset.** The information in this table is collected from the following references: ArNat [2,3], NatA [4–7], NatB [8–10] , NatD [11,12], NatE [13–15], NatF [16,17], NatH [18,19], RimI [20,21], RimJ [20,22], RimL [23,24].

| **Group** | **Name of the complex** | **Specificity** | **Assembly** | **Modification** | **Localization** |
| --- | --- | --- | --- | --- | --- |
|  |  |  |  |  |  |
| **ArNat** | ssNAT | Ser | Monomeric | Co-translationnal | Nucleus |
|  |  | Met-aa2 |  |  |  |
| **Naa10** | Nat A | Ser, Ala, Thr, Gly, Val | Complexed with Naa15 | Co-translationnal | Nucleus |
|  |  | Glu, Asp | Monomeric | Post-translational | Cytosol |
| **Naa20** | Nat B | Met (-Asp, -Glu, -Asn, -Gln) | Complexed with Naa25 | Co-translational | Nucleus |
| **Naa40** | Nat D | Ser | Monomeric | Co-translationnal | Nucleus |
| **Naa50** | Nat E | Met (-Leu, -Lys, -Ala, -Met) | Complexed with NatA | Co-translationnal | Nucleus |
| **Naa60** | Nat F | Met (-Lys, -Ala, -Val, -Met) | Monomeric | Post-translational | Membrane of the Golgi |
| **Naa80** | Nat H | Glu, Asp | Monomeric | Post-translational | Processed actin |
| **RimI** | RimI | Ala | Monomeric | Co-translationnal | Nucleus |
| **RimJ** | RimJ | Ser | / | Co-translationnal | Nucleus |
| **RimL** | RimL | Ser | Dimeric | Co-translationnal | Nucleus |

[1] Foyn H, Jones JE, Lewallen D, Narawane R, Varhaug JE, Thompson PR, et al. Design, synthesis, and kinetic characterization of protein N-terminal acetyltransferase inhibitors. ACS Chem Biol 2013;8:1121–7. https://doi.org/10.1021/cb400136s.

[2] Mackay DT, Botting CH, Taylor GL, White MF. An acetylase with relaxed specificity catalyses protein N-terminal acetylation in Sulfolobus solfataricus. Mol Microbiol 2007;64:1540–8. https://doi.org/10.1111/j.1365-2958.2007.05752.x.

[3] Liszczak G, Marmorstein R. Implications for the evolution of eukaryotic amino-terminal acetyltransferase (NAT) enzymes from the structure of an archaeal ortholog. Proc Natl Acad Sci U S A 2013;110:14652–7. https://doi.org/10.1073/pnas.1310365110.

[4] Arnesen T, Anderson D, Baldersheim C, Lanotte M, Varhaug JE, Lillehaug JR. Identification and characterization of the human ARD1-NATH protein acetyltransferase complex. Biochem J 2005;386:433–43. https://doi.org/10.1042/BJ20041071.

[5] Arnesen T, Van Damme P, Polevoda B, Helsens K, Evjenth R, Colaert N, et al. Proteomics analyses reveal the evolutionary conservation and divergence of N-terminal acetyltransferases from yeast and humans. Proc Natl Acad Sci U S A 2009;106:8157–62. https://doi.org/10.1073/pnas.0901931106.

[6] Van Damme P, Evjenth R, Foyn H, Demeyer K, De Bock P-J, Lillehaug JR, et al. Proteome-derived Peptide Libraries Allow Detailed Analysis of the Substrate Specificities of N ^α^ -acetyltransferases and Point to hNaa10p as the Post-translational Actin N ^α^ -acetyltransferase. Mol Cell Proteomics 2011;10:M110.004580. https://doi.org/10.1074/mcp.M110.004580.

[7] Liszczak G, Goldberg JM, Foyn H, Petersson EJ, Arnesen T, Marmorstein R. Molecular basis for N-terminal acetylation by the heterodimeric NatA complex. Nat Struct Mol Biol 2013;20:1098–105. https://doi.org/10.1038/nsmb.2636.

[8] Polevoda B, Cardillo TS, Doyle TC, Bedi GS, Sherman F. Nat3p and Mdm20p are required for function of yeast NatB Nα-terminal acetyltransferase and of actin and tropomyosin. J Biol Chem 2003;278:30686–97. https://doi.org/10.1074/jbc.M304690200.

[9] Starheim KK, Arnesen T, Gromyko D, Ryningen A, Varhaug JE, Lillehaug JR. Identification of the human N α -acetyltransferase complex B (hNatB): a complex important for cell-cycle progression. Biochem J 2008;415:325–31. https://doi.org/10.1042/BJ20080658.

[10] Van Damme P, Lasa M, Polevoda B, Gazquez C, Elosegui-Artola A, Kim DS, et al. N-terminal acetylome analyses and functional insights of the N-terminal acetyltransferase NatB. Proc Natl Acad Sci 2012;109:12449–54. https://doi.org/10.1073/pnas.1210303109.

[11] Song OK, Wang X, Waterborg JH, Sternglanz R. An Nalpha-acetyltransferase responsible for acetylation of the N-terminal residues of histones H4 and H2A. J Biol Chem 2003;278:38109–12. https://doi.org/10.1074/jbc.C300355200.

[12] Magin RS, Liszczak GP, Marmorstein R. The molecular basis for Histone H4- and H2A-specific amino-terminal acetylation by NatD. Structure 2015;23:332–41. https://doi.org/10.1016/j.str.2014.10.025.

[13] Arnesen T, Anderson D, Torsvik J, Halseth HB, Varhaug JE, Lillehaug JR. Cloning and characterization of hNAT5/hSAN: An evolutionarily conserved component of the NatA protein N-α-acetyltransferase complex. Gene 2006;371:291–5. https://doi.org/10.1016/j.gene.2005.12.008.

[14] Evjenth R, Hole K, Karlsen OA, Ziegler M, Amesen T, Lillehaug JR. Human Naa50p (Nat5/San) displays both protein Nα- and Nε-acetyltransferase activity. J Biol Chem 2009;284:31122–9. https://doi.org/10.1074/jbc.M109.001347.

[15] Van Damme P, Hole K, Gevaert K, Arnesen T. N-terminal acetylome analysis reveals the specificity of Naa50 (Nat5) and suggests a kinetic competition between N-terminal acetyltransferases and methionine aminopeptidases. Proteomics 2015;15:2436–46. https://doi.org/10.1002/pmic.201400575.

[16] Aksnes H, Goris M, Strømland Ø, Drazic A, Waheed Q, Reuter N, et al. Molecular determinants of the N-Terminal acetyltransferase Naa60 anchoring to the Golgi membrane. J Biol Chem 2017;292:6821–37. https://doi.org/10.1074/jbc.M116.770362.

[17] Aksnes H, Van Damme P, Goris M, Starheim KK, Marie M, Støve SI, et al. An organellar nα-acetyltransferase, naa60, acetylates cytosolic n termini of transmembrane proteins and maintains golgi integrity. Cell Rep 2015;10:1362–74. https://doi.org/10.1016/j.celrep.2015.01.053.

[18] Goris M, Magin RS, Foyn H, Myklebust LM, Varland S, Ree R, et al. Structural determinants and cellular environment define processed actin as the sole substrate of the N-terminal acetyltransferase NAA80. Proc Natl Acad Sci 2018:201719251. https://doi.org/10.1073/pnas.1719251115.

[19] Drazic A, Aksnes H, Marie M, Boczkowska M, Varland S, Timmerman E, et al. NAA80 is actin’s N-terminal acetyltransferase and regulates cytoskeleton assembly and cell motility. Proc Natl Acad Sci 2018:201718336. https://doi.org/10.1073/pnas.1718336115.

[20] Yoshikawa A, Isono S, Sheback A, Isono K. Cloning and nucleotide sequencing of the genes rimI and rimJ which encode enzymes acetylating ribosomal proteins S18 and S5 of Escherichia coli K12. MGG Mol Gen Genet 1987;209:481–8. https://doi.org/10.1007/BF00331153.

[21] Vetting MW, Bareich DC, Yu M, Blanchard JS. Crystal structure of RimI from Salmonella typhimurium LT2, the GNAT responsible for N(alpha)-acetylation of ribosomal protein S18. Protein Sci 2008;17:1781–90. https://doi.org/10.1110/ps.035899.108.

[22] Roy-Chaudhuri B, Kirthi N, Kelley T, Culver GM. Suppression of a cold-sensitive mutation in ribosomal protein S5 reveals a role for RimJ in ribosome biogenesis. Mol Microbiol 2008;68:1547–59. https://doi.org/10.1111/j.1365-2958.2008.06252.x.

[23] Tanaka S, Matsushita Y, Yoshikawa A, Isono K. Cloning and molecular characterization of the gene rimL which encodes an enzyme acetylating ribosomal protein L12 of Escherichia coli K12. Mol Gen Genet 1989;217:289–93. https://doi.org/10.1007/BF02464895.

[24] Vetting MW, De Carvalho LPS, Roderick SL, Blanchard JS. A novel dimeric structure of the RimL Na-acetyltransferase from Salmonella typhimurium. J Biol Chem 2005;280:22108–14. https://doi.org/10.1074/jbc.M502401200.

**Figure S1. Heatmap representation of the pairwise Root Mean Square Deviations (RMSD) for representative structures.** The dendrogram reflects the hierarchical clustering based on the RMSD values. The heatmap color scale goes from red (0Å < RMSD < 1Å; structural similarity) to yellow (3Å < RMSD < 4Å). Names of enzymes from eukaryotes are highlighted in blue, those of bacteria in purple and green is used for archaeal NATs.


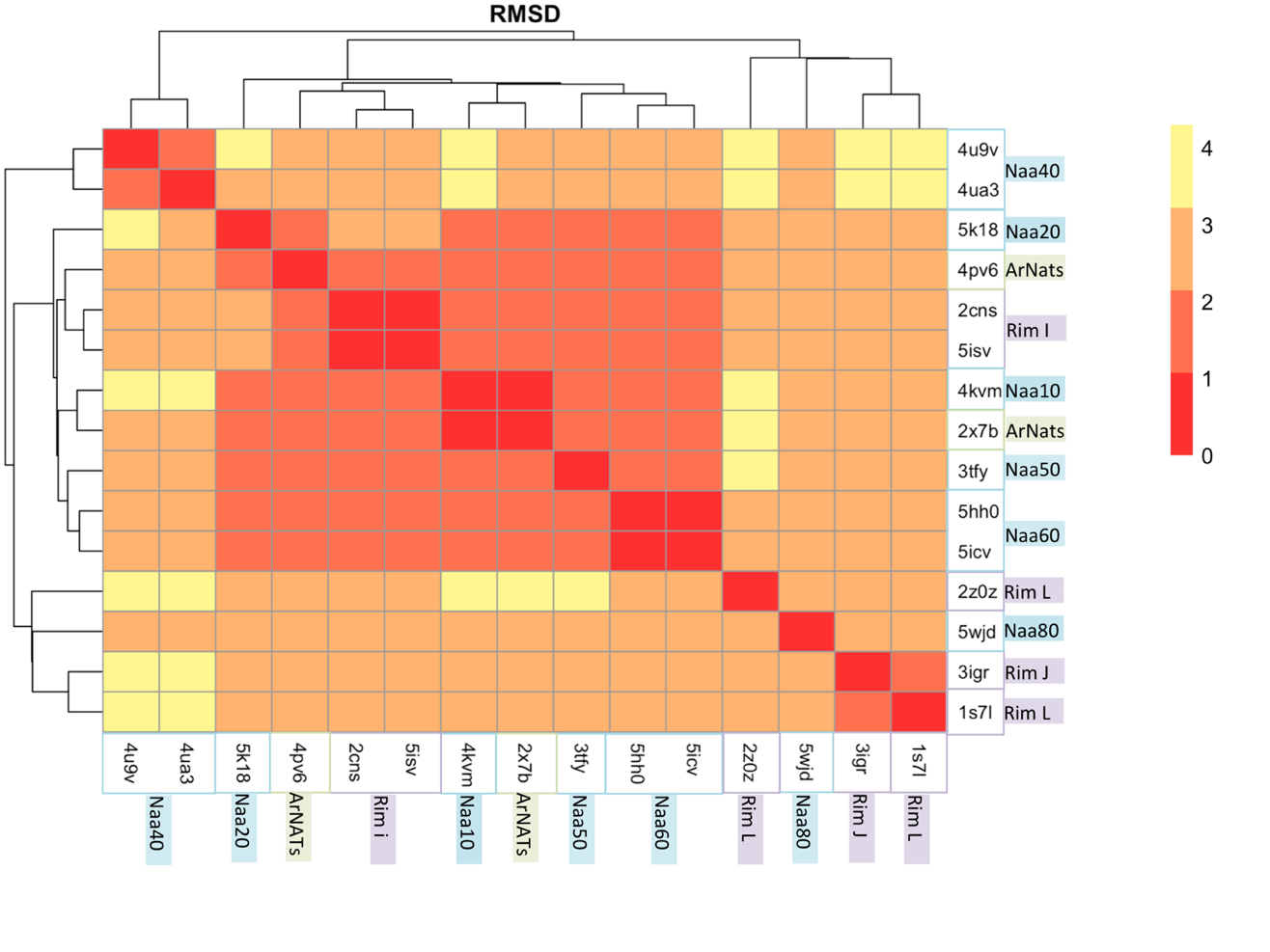


**Figure S2. Effect of the presence of a cofactor or a bisubstrate on the normalized fluctuations of the Naa10 from *Saccharomyces pombe*.** Naa10 from *S. pombe* has been crystallized with Naa15 (PDB id: 4kvm and 4kvo) and without Naa15 (PDB id: 4kvx).The main difference between the structures of the two states is a rearrangement of helix α2 and of the α1α2 loop. The X-ray structures of the Naa10-Naa15 complex contain either a bisubstrate inhibitor (4kvm) or only a cofactor (4kvo). The structure of the uncomplexed Naa10 form has only the cofactor (4kvx). Here we extract the cartesian coordinates of the catalytic domains only (Naa10) and compare the fluctuation profiles in different conditions. We compute the fluctuations on Naa10 in the presence of the co-factor (for 4kvo and 4kvx) or bisubstrate (4kvm) and on the structures after removal of the co-factor (4kvo, 4kvx) or bisubstrate (4kvm). The amplitude of the fluctuations of the loops is modulated by the bisubstrate or cofactor and by the position of the α1α2 region but the three loops β2β3, β3β4 and β6β7 remain the most flexible regions of the Naa10 structure.

**Figure S3. Correlations maps for the representative of each NAT group.** Dark red and blue show correlated motions between pairs of residues (close to 1 or -1, respectively), when lighter colours refer to correlations close to 0. Long-range correlations are found within two blocks highlighted by the green and pink frames. For all the NATs the highest correlations are found within these blocks and not in-between.


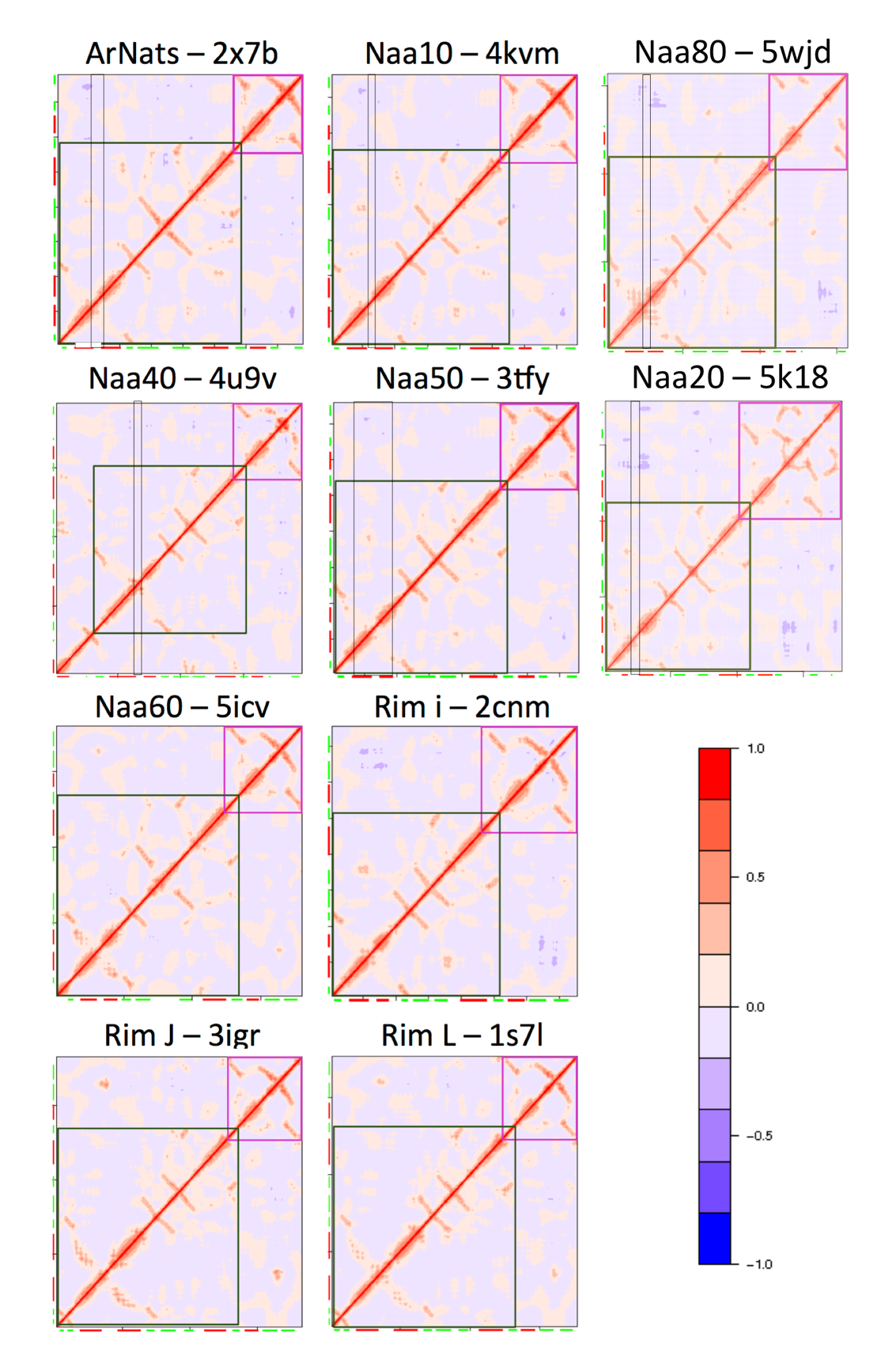


**Figure S4. Vector fields of the six lowest frequency normal modes of the human NATs. The following representative structures are chosen: (A)** Naa10 (PDB ID: **4KVM**), **(B)** Naa20 (PDB ID: **5K18**), **(C)** Naa40 (PDB ID: **4U9V**), **(D)** Naa60 (PDB ID: **5ICV**), and **(E)** Naa80 (PDB ID: **5WJD**). The protein is represented with a cartoon representation and the surface of the two subdomains is displayed in either green or pink. The normal mode vectors are represented by arrows indicating the directions of motion. Red and blue colors depict positive and negative directions and are chosen arbitrarily.

**(A)**

**(B)**

**(C)**

**(D)**

**(E)**

**Figure S5. Ligand binding sites of the human NATs in native structure and models generated along the six lowest frequency normal modes.** The structures of **(A)** Naa10 (PDB ID: **4KVM**), **(B)** Naa20 (PDB ID: **5K18**), **(C)** Naa40 (PDB ID: **4U9V**), **(D)** Naa60 (PDB ID: **5ICV**), and **(E)** Naa80 (PDB ID: **5WJD**) are represented in cartoons with light blue β-strands and orange α-helices. For each mode (vertical panel), the middle structure depicts the initial X-ray structure and the upper and lower structures, the corresponding mode deformations in negative and positive directions respectively. The red surface represents the tunnel calculated by CAVER Analyst (see Materials and Methods). For a given mode, the right-most images show a front view of the protein and its cavity clipped by the plane indicated by the black vertical line on the left-most side view. The cavity constriction is noticeable with the blur effect inside the cavity surface on the front view.

**(A)**

**(B)**

**(C)**

**(D)**

**(E)**

1. [↑](#endnote-ref-1)
